# Association between Tetrodotoxin Resistant Channels and Lipid Rafts Regulates Sensory Neuron Excitability

**DOI:** 10.1371/journal.pone.0040079

**Published:** 2012-08-01

**Authors:** Alessandro Pristerà, Mark D. Baker, Kenji Okuse

**Affiliations:** 1 Division of Cell and Molecular Biology, Faculty of Natural Sciences, Imperial College London, London, United Kingdom; 2 Neuroscience and Trauma Centre, Blizard Institute, Queen Mary University of London, Barts and The London School of Medicine and Dentistry, London, United Kingdom; University of Waterloo, Canada

## Abstract

Voltage-gated sodium channels (VGSCs) play a key role in the initiation and propagation of action potentials in neurons. Na_V_1.8 is a tetrodotoxin (TTX) resistant VGSC expressed in nociceptors, peripheral small-diameter neurons able to detect noxious stimuli. Na_V_1.8 underlies the vast majority of sodium currents during action potentials. Many studies have highlighted a key role for Na_V_1.8 in inflammatory and chronic pain models. Lipid rafts are microdomains of the plasma membrane highly enriched in cholesterol and sphingolipids. Lipid rafts tune the spatial and temporal organisation of proteins and lipids on the plasma membrane. They are thought to act as platforms on the membrane where proteins and lipids can be trafficked, compartmentalised and functionally clustered. In the present study we investigated Na_V_1.8 sub-cellular localisation and explored the idea that it is associated with lipid rafts in nociceptors. We found that Na_V_1.8 is distributed in clusters along the axons of DRG neurons *in vitro* and *ex vivo.* We also demonstrated, by biochemical and imaging studies, that Na_V_1.8 is associated with lipid rafts along the sciatic nerve *ex vivo* and in DRG neurons *in vitro.* Moreover, treatments with methyl-β-cyclodextrin (MβCD) and 7-ketocholesterol (7KC) led to the dissociation between rafts and Na_V_1.8. By calcium imaging we demonstrated that the lack of association between rafts and Na_V_1.8 correlated with impaired neuronal excitability, highlighted by a reduction in the number of neurons able to conduct mechanically- and chemically-evoked depolarisations. These findings reveal the sub-cellular localisation of Na_V_1.8 in nociceptors and highlight the importance of the association between Na_V_1.8 and lipid rafts in the control of nociceptor excitability.

## Introduction

Voltage-gated sodium channels (VGSCs) are necessary for the generation and propagation of action potentials [1]. Three of the VGSCs isoforms encoded in mammals are TTX resistant (TTX-r): Na_V_1.5 (expressed in the cardiac tissue [2], Na_V_1.8 and Na_V_1.9, both expressed in nociceptors. More specifically, Na_V_1.8 is expressed in unmyelinated, C-type, small-diameter sensory neurons [3]. These cells are responsible for the detection of noxious stimuli and play a major role in the hyperalgesia and allodynia that accompany chronic pain states [4]. Electrophysiological studies in C-fibre cell bodies have demonstrated that Na_V_1.8 is essential for normal electrogenesis, underlying the vast majority (>85%) of the inward current that ﬂows during the upstroke of action potentials [5]. On the other hand Na_V_1.9 does not contribute to action potentials but is involved in setting the resting membrane potential [6].

Given the key electrophysiological features of Na_V_1.8 and its restricted expression, pharmacological and genetic studies on knock-out mice have been carried out to investigate its role in inflammatory and chronic pain states. Indeed it has been found that Na_V_1.8 play a key role in both inflammatory and neuropathic pain conditions [7], [8], [9].

Unlike the case of myelinated fibres, which show restricted localisation of VGSCs at nodes of Ranvier and axon initial segments, the precise localisation of VGSCs in unmyelinated fibres has not been investigated in detail, and the sub-cellular localisation of Na_V_1.8 still remain uncharacterised. It is believed that VGSCs are uniformly distributed in unmyelinated fibres, based on indirect evidences such as continuous conduction and homogeneous appearance of freeze fracture photomicrographs [10]. Some reports suggest that Na_V_1.8 is found along the entire length of the unmyelinated sensory axons in the cornea [11] and the sciatic nerve [12], although the same group has shown that Na_V_1.8 appeared to be in clusters at the varicosities and inter-connecting regions of the nerve terminals in unmyelinated axons of intraepidermal fibres [13].

Lipid rafts are defined as “dynamic, nanoscale, sterol–sphingolipids enriched, ordered assemblies of proteins and lipids” [14], [15], [16]. Compared to the liquid disordered state of the bulk membrane they exist in a liquid ordered state and they are resistant to non-ionic detergent lysis at 4°C [17], [18]. The core role of lipid rafts is to laterally organise the cell membrane, both spatially and temporally. They act as hubs on the cellular membrane where proteins can be sorted and functionally localised. Lipid rafts regulate the trafficking, clustering and electrophysiological properties of ion channels. Overall, they contribute to shape cell membrane excitability (Reviewed in [19]). For example, hippocampal neurons contain lipid rafts in the dendrites, and rafts are important for the stabilisation of AMPA receptor clusters [20]. Potassium channel K_V_2.1 is also associated with lipid rafts, and rafts directly modulate its electrophysiological features [21].

Several studies have directly investigated the membrane trafficking of Na_V_1.8 (reviewed in [22]. However, in this study we focussed on Na_V_1.8 localisation and association with lipid rafts in DRG neurons. We found that Na_V_1.8 is normally localised in clusters along the axons of unmyelinated neurons and that it associates with lipid rafts. Importantly, the disruption of lipid rafts in DRG neurons led to the shift of Na_V_1.8 into the non-raft portion of the membrane and this redistribution correlated with impaired neuronal excitability.

## Materials and Methods

### Cell culture

DRGs from female Wistar rats (150 grams) were harvested in cold DMEM and any excess dorsal roots and spinal nerves were trimmed under a stereo microscope. DRGs were incubated with 0.125% Collagenase XI (Sigma) and 0.1 mg/ml DNase II (Sigma) in DMEM for 90 min at 37°C. After enzymatic digestion, DRGs were triturated with a cut 1 ml tip until a cell suspension was obtained. Cells were spun down, resuspended in pre-warmed DMEM and filtered through a 70 µm mesh (BD Biosciences). DRG neurons were recovered by using 10% BSA (PAA)/ DMEM cushions and plated on 13 mm glass coverslips coated with poly-L-lysine and laminin in complete media (DMEM, 10% fetal bovine serum (FBS), penicillin/streptomycin (1∶100; Sigma), NGF (50 ng/ml; Peprotech) and aphidicolin (10 µM; Sigma). 10,000 and 150,000 cells per coverslips were plated for immunofluorescence and biochemistry purposes, respectively. Cells were maintained in a 95% air/5% CO_2_ humidified incubator.

### Immunofluorescence and GM1 detection on cultured DRG neurons

DRG neurons were washed in PBS (Gibco; Invitrogen) and fixed with 4% paraformaldehyde (PFA) for 10 min at room temperature (RT). Following washes in PBS, DRG neurons were incubated with primary antibodies. All the antibodies were diluted in 10% goat serum (GS) for 1 hour at RT at the following dilution: rabbit anti Na_V_1.8 [23], [24] 1∶200, mouse anti-Peripherin (Chemicon) 1∶200, mouse anti-NF200 (Chemicon) 1∶500. After washes cells were incubated with fluorescently labeled secondary antibodies, diluted in 10% GS for 1 hour at RT. When nuclear counterstain was needed, cells were incubated with Hoechst 33342 (Invitrogen) diluted 1∶10000 in PBS for 10 min at RT and subsequently washed in PBS. Coverslips were mounted on glass slides with anti fade agent AF1 (Citifluor LTD) and sealed with nail varnish.

To detect GM1 ganglioside DRG neurons were washed with PBS and incubated with 1 µg/ml biotinylated cholera toxin β subunit (CTB; Invitrogen) in PBS for 20 min at RT. After washes with PBS, DRG neurons were incubated with 1∶1000 streptavidin-488 (Invitrogen) for 20 min at RT. Cells were washed in PBS, fixed with 4% PFA (Sigma) for 10 min at RT. and processed for immunofluorescence.

Samples were analysed on a wide-field Nikon 80i microscope and pictures acquired with an ultrahigh-quality Nikon DXM1200F digital camera controlled with LUCIA G software.

### Electroporation of DRG neurons

Exogenous DNA was delivered into DRG neurons by electroporation with a Neon transfection system (Invitrogen). Electroporation was carried out before plating the cells. The cell suspension was washed in PBS and spun down. The pellet was resuspended in 27 µl buffer R (Invitrogen) plus 3 µl of plasmid DNA (concentration of plasmid DNA higher than 1 µg/µl). The cell suspension was aspirated in a 10 µl tip and the electric pulses delivered (2 pulses, 1200 volts, 20 msec). For electroporation purposes, 200,000 cells were electroporated in four rounds of electroporation. After the electric pulses neurons were plated in pre-warmed DMEM with 10% FBS without antibiotics. Two hours after plating, NGF was added to the cells. The day after plating, media was replaced with complete DMEM and media changed every two days.

### Teased fibre preparation from sciatic nerve and immunofluorescence

Sciatic nerves were harvested and placed on top of a 2% gelatin (Sigma) coated glass coverslip. Under a stereomicroscope nerve fascicles were freed from the epineurium by pulling them with fine tips tweezers. The fascicles were dissociated in fibre bundles and single fibres by gently pulling them with forceps. The sciatic nerve fibres were washed in PBS and fixed in 4% PFA. After washes fibres were permeabilised with 0.1% Triton X-100 in PBS 10 min at RT. Following washes, samples were blocked in 10% GS in PBS for 30 min at RT and incubated with primary antibodies diluted in 10% GS/PBS for 2 hours at RT. (rabbit anti Na_V_1.8 1∶100, mouse anti Peripherin 1∶100). After washes with PBS samples were incubated with fluorescently labelled secondary antibodies diluted in 10% GS in PBS for 1 hour at RT.

### Lipid raft purification

35 DRGs and sciatic nerves (left and right; 70 mg) were dissected from female Wistar rats (150 grams) and homogenised, by using a glass pestle and mortar, in homogenisation buffer (150 mM NaCl, 5 mM dithiothreitol, 5 mM EDTA, 25 mM Tris-HCl, supplemented with 1∶1000 protease inhibitor cocktail set III (Merck), pH 7.4). The homogenate was centrifuged at 3600 rpm for 10 min at 4°C to pellet chromatin, the supernatant recovered and adjusted with Triton X-100 (Sigma) to give a final concentration of 1% Triton X-100. When DRG cultures were used, neurons were recovered by scraping the cells in homogenisation buffer supplemented with 1% Triton X-100.

Lipid rafts were purified by incubating the samples for 30 min on ice. The lysate was mixed with 60% OptiPrep (iodixanol; Sigma) to obtain a final concentration 40% OptiPrep. The 40% fraction was layered in a 5/16×1 3/8 inch ultracentrifuge tube (Beckman) with 30% and 0% OptiPrep layers prepared in homogenisation buffer with 1% Triton X-100.

Samples were finally centrifuged at 36000 rpm for 4.5 hours and after centrifugation the whole gradient was recovered from the tubes. All procedures were carried out at 4°C, with pre-cooled solutions.

### Immunoblotting

Equal volumes of the fractions recovered from ultracentrifugation were subjected to SDS-PAGE. After electrophoresis proteins were transferred onto PVDF membrane (Amersham) and non specific sites were blocked with 5% dried fat-free milk dissolved in PBS supplemented with 0.1% Tween-20 (Sigma) (PBS-T) over-night (ON) at 4°C.

After washes in PBS-T membranes were incubated with the following primary antibodies: rabbit anti Na_V_1.8 [23], [24], mouse anti Flotillin-1 (DB Biosciences), mouse anti transferrin receptor (Zymed; Invitrogen). All antibodies were used 1∶1000 for 1 hour at RT. Following washes the membranes were incubated with the appropriate secondary antibodies conjugated to horseradish peroxidase (HRP) diluted in 10% GS in PBS-T.

For dot blot analysis, 1 µl of each layer recovered from the centrifugation step was applied to a nitrocellulose membrane (0.45 μm; Amersham) and dried at RT. Non specific interactions were blocked by incubating the membranes with 5% BSA diluted in PBS for 1 hour at RT. Membrane was probed with biotinylated CTB at a final concentration of 0.1 µg/ml in PBS for 20 min and, after three washes, was incubated with HRP conjugated Streptavidin (Dako) diluted 1∶10,000 in PBS for 20 min.

Signals were developed with the enhanced chemiluminescence detection system kit (Applichem) and detected with Fujifilm LAS-3000 Imaging System.

When needed, membranes were stripped by incubating them in stripping buffer (2% SDS, 100 mM β-mercaptoethanol added fresh, 50 mM Tris-HCl, pH 6.8) at 50°C for 30 min with gentle shaking. Membranes were subsequently thoroughly washed in PBS-T and blocked overnight in 5% dried fat-free milk dissolved in PBS-T before immunoblotting.

### Sterol complexes preparation

7-ketocholesterol (7KC; Sigma) and cholesterol (Sigma) were complexed with methyl-β-cyclodextrin (MβCD) (Sigma) as previously described in literature [25]. Briefly, sterols were dissolved in 96% ethanol to a final concentration of 15 mg/ml. MβCD was dissolved in sterile water to a final concentration of 50 mg/ml. 400 μl of 50 mg/ml MβCD was heated to 80°C and 4×10 μl of 15 mg/ml sterols added every 5 min. This preparation led to stock sterols solutions (3.4 mM 7KC, 3.5 mM cholesterol). The compounds were prepared fresh on the day of the experiment.

### Fluo-4 AM loading and imaging

DRG neurons were washed three times with Normal solution (140 mM NaCl, 5 mM KCl, 1.8 mM CaCl_2_, 2 mM MgCl_2_, 10 mM D-Glucose, 10 mM HEPES, pH 7.4) and incubated with 4 μM Fluo-4 AM diluted in Normal solution for 30 min at RT in the dark. After three washes in Normal solution, cells were left 30 min at RT for the de-esterification step. Following three washes neurons were imaged on a Leica SP5 inverted confocal microscope at 37°C in Normal solution. Fluo-4 was excited with a 488 nm wavelength and emitted fluorescence was detected in the range 500–570 nm.

### Mechano-stimulation

Mechano-stimulation was performed under manual visual control using a Leica SP5 inverted confocal microscope equipped with micro-manipulator Inject Man N1 2 (Eppendorf). The motorised head of the micro-manipulator was set at 45° against the main surface of the culture wells and mechano-stimulation of the neurons was achieved by using a fine glass probe with a tip diameter of 1.0 µm (Femtotip; Eppendorf). All the experiments were performed in Normal solution in a controlled temperature incubator set at 37°C.

We probed the neurons three times and for our analysis we defined a neuron as “responsive” if the axonal stimulation evoked a soma response after one of these three stimulations. In case of multiple responses only the first stimulation was included in the analysis, to minimise the effect of sensitisation/desensitisation. Also, the increase of fluorescence at the level of the cell body had to be higher than 10% (threshold set subjectively) compared to baseline fluorescence to be classified as “response”, and had to reach the maximum level within 20 seconds. When a neuron was found to be responsive, the time point of maximum neurite displacement was considered to be the time of probe “contact”. Cells that showed swelling or rupture of the axons upon mechanic stimulation were discarded from the analysis.

### Campenot set-up and chemical stimulation

Campenot chambers were custom designed and fabricated with Teflon (Tyler Research Corporation). The chambers were set-up the day before plating the neurons. Briefly, 35 mm plastic dishes (BD Falcon) were scratched with a pin-rake in the middle portion and the scratched region was coated with 0.1 mg/ml poly-L-lysine. The middle portion of the scratched region was overlaid with 30 µl of 1% methylcellulose (Sigma), 10 µg/ml laminin (Invitrogen), 50 ng/ml NGF diluted in DMEM with antibiotics. The bottom surface of the Teflon dividers was greased with autoclaved high-vacuum grease (Dow Corning). The dividers were sealed to the plastic dishes by turning upside-down the dish-chamber complex and by applying gentle pressure to the plastic dishes with a fine forceps. The assembled Campenot chambers were left 2 hours at 37°C to equilibrate. 200 µl of DMEM was applied to the side chambers and left over-night at 37°C to test for leakage. The following day any leaky chambers were discarded.

Chambers were washed three times with pre-warmed DMEM, and coated with 10 µg/ml laminin. 50,000 DRG neurons were plated in DMEM without NGF and supplemented with 10% FBS, antibiotics, 10 µM aphidicolin. Axonal outgrowth was promoted by NGF diluted in 10% FBS DMEM with antibiotics and 10 µM aphidicolin. Experiments were carried out 14 days after plating, when extensive neurite outgrowth in the furthest chamber was obtained.

The day of the experiment DRG neurons were loaded with Fluo-4-AM as described above. For imaging purposes all chambers were filled with 150 µl of Normal solution, and axon endings were chemically stimulated with 10 µM capsaicin (Fluka), 10 µM bradykinin (Sigma) and 300 µM ATP (Sigma) diluted in Normal solution. Cell bodies were visualised with a 10x air objective on a Leica SP5 confocal microscope equipped with a heated chamber maintained at 37°C.

Neurons that showed a transient increase of fluorescence higher than 3% above baseline upon chemical stimulation, were classified as responsive. This value was chosen as it represents at least three times the increase that occurred in a few control cells following vehicle application (1% increase in 1 out of 58 cells). Also, the increase of fluorescence had to show a transient profile to be classified as positive. Cells showing an oscillation in fluorescence before the chemical stimulation were excluded from the analysis.

## Results

### Na_V_1.8 localises in clusters along the axons of cultured small diameter DRG neurons

**Figure 1:**
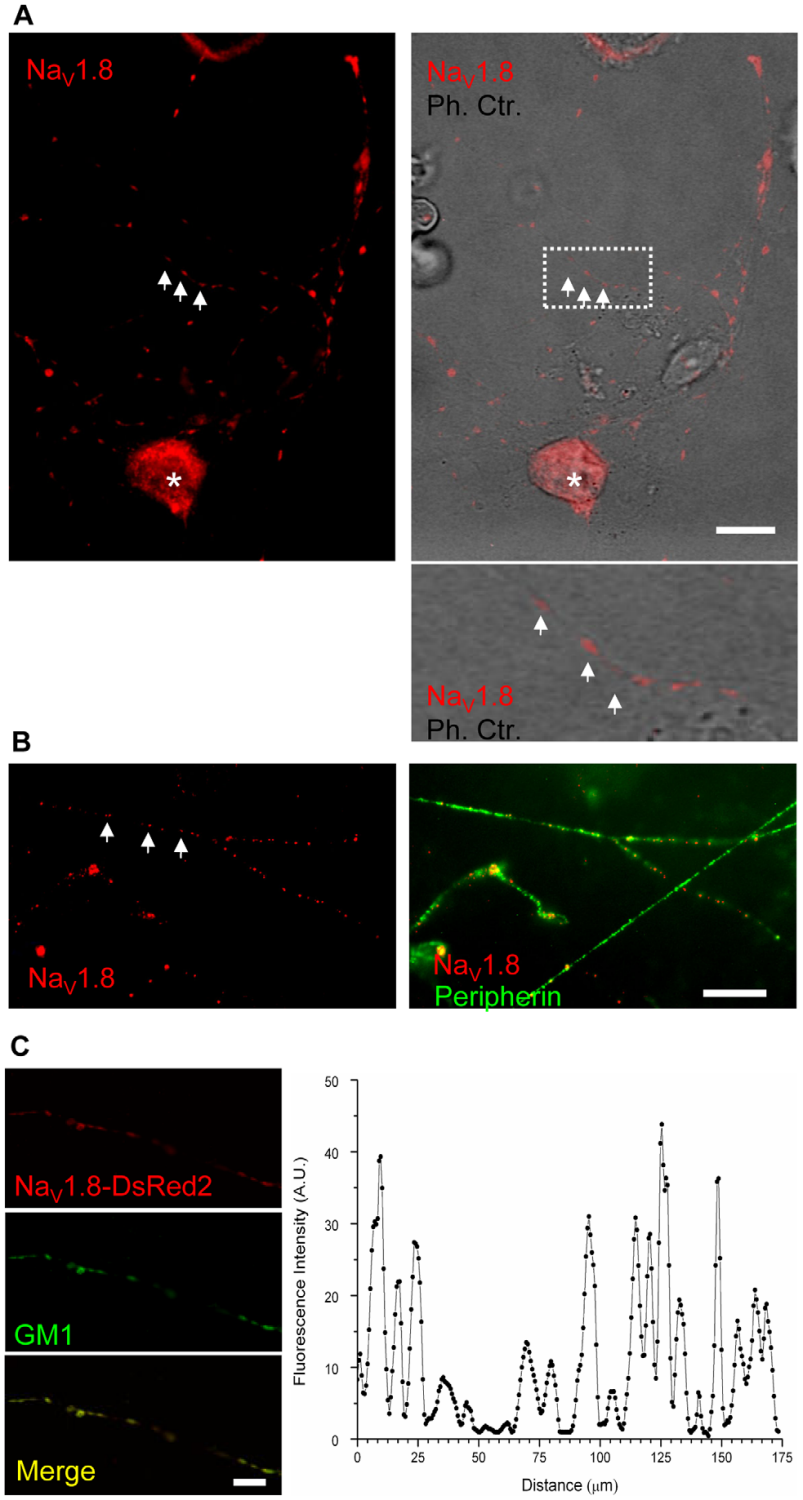
Na_V_1.8 is distributed in clusters along the axons of small, unmyelinated DRG neurons *in vitro*. Endogenous Na_V_1.8 was immuno-localised in cultured DRG neurons after 2 DIV. Small-diameter neurons were identified by morphology (A, right panel) and by the immuno-reactivity for Peripherin (B, right panel). The region framed by the dotted square in A is magnified in the inset below. Na_V_1.8 is distributed in distinct puncta along the neurites, of small-diameter neurons (A, B; arrows pinpoint example of clusters, which are distributed throughout the neurites). Na_V_1.8 was also found to be enriched at the level of the cell bodies (Figure 1A, asterisk). The fluorescent construct Na_V_1.8-DsRed2 was visualised in DRG neurons. The image shows Na_V_1.8-DsRed2 distributed in clusters along the axon of DRG neuron (C, left panel). The discontinuous distribution of the fluorescent construct has been mapped by quantifying pixel intensity along the neurite (C, graph; right panel). Also, the fluorescent construct Na_V_1.8-DsRed2 colocalises with GM1 puncta along the neurite of DRG neurons, as shown by the superimposed images of Na_V_1.8-DsRed2 and GM1 (merge). Scale bars are 20 μm.

We first analysed the sub-cellular distribution of Na_V_1.8 in DRG neurons *in vitro* by immunocytochemistry. After two days *in vitro* (DIV), DRG neurons showed an extensive neurite outgrowth and we found that, in small-diameter cell body neurons (<25 µm), most likely to be nociceptors [26], [27], Na_V_1.8 was expressed both at the level of the cell body (Figure 1A, asterisk) and along the neurites, where in the latter location it was distributed in a clustered fashion (Figure 1A, arrows; mean length of the clusters ± SEM  = 3.38±0.40 μm; n = 3; Total clusters counted  = 255). The phase contrast image in Figure 1A, and the magnified inset, show that Na_V_1.8 immunoreactivity was associated with intact neurons, and that the clustered appearance in small-diameter neurons was not due to uneven morphology of the axons (e.g. rupture due to necrosis, apoptosis). To further confirm Na_V_1.8 sub-cellular distribution *in vitro* we performed double immunocytochemistry between Na_V_1.8 and Peripherin (a marker of small-diameter, unmyelinated nociceptors). In agreement with the findings for neurons discriminated only in terms of their cell body diameters, Na_V_1.8 was found to be localised along the neurites in distinguishable puncta in Peripherin-positive small-diameter neurons (Figure 1B, arrows). We found also that a minor population of large-diameter neurons expressed Na_V_1.8. In these cells Na_V_1.8 was either evenly distributed or associated in large patches (Figure S1 A, B).

To eliminate the possibility that the clusters of Na_V_1.8 were due to artificial aggregation elicited by the antibody, we analysed the Na_V_1.8 sub-cellular localisation by using a fluorescent version of Na_V_1.8. We cloned the fluorescent tag DsRed2 to the C-terminus of Na_V_1.8 to create the fusion protein Na_V_1.8-DsRed2 under the influence of CMV immediate early promoter. By mean of electroporation we delivered the plasmid DNA into the DRG neurons and monitored Na_V_1.8-DsRed2 fluorescence. We found that Na_V_1.8-DsRed2 showed a clustered localisation in neurons along the axons (Figure 1C, left panel), similar to that shown by the antibody-based technique we previously described. Densitometric analysis of Na_V_1.8-DsRed2 fluorescence along the axon of the DRG neuron showed that clear peaks of fluorescence appeared along the axon length (Figure 1C, right panel).

**Figure 2:**
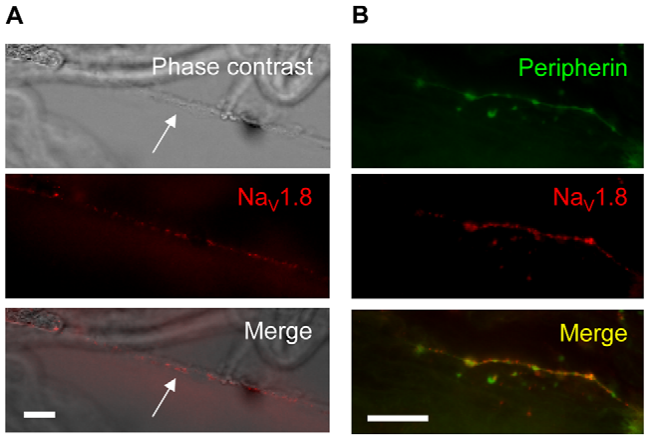
Na_V_1.8 is distributed in clusters along the axons of small, unmyelinated DRG neurons *in vivo*. Na_V_1.8 is clustered in puncta along the unmyelinated fibres of rat sciatic nerve. Teased unmyelinated fibres were identified by morphology (A, arrow) and by immuno-reactivity for Peripherin (B). Scale bars are 20 μm.

We also investigated Na_V_1.8 distribution along the axons of small-diameter, unmyelinated fibres *ex vivo*. In small-diameter fibres, identified both by low contrast in bright-field imaging (Figure 2A, arrow) and by positive immunolabelling for Peripherin (Figure 2B), Na_V_1.8 showed a clustered distribution (Figure 2A, B), similar to that identified in the *in vitro* preparations.

### Na_V_1.8 clusters co-localise with lipid raft markers in DRG neurons *in vitro*

Our findings indicated that Na_V_1.8 was distributed in a punctuate fashion in the processes of unmyelinated neurons, and we hypothesised that it localises within the membrane micro-domains known as lipid rafts. Lipid rafts have already been described as microdomains in which sub-populations of specialised proteins are known to cluster [28], [29]. Lipid rafts by definition have a distinctive lipid make-up, where ganglioside GM1 is highly enriched and is a standard marker of these micro-domains [30]. To investigate the association between Na_V_1.8 and lipid rafts *in vitro* we have visualised gangliosides GM1 by Cholera Toxin B subunit (CTB), and localised Na_V_1.8 by immunocytochemistry.

**Figure 3:**
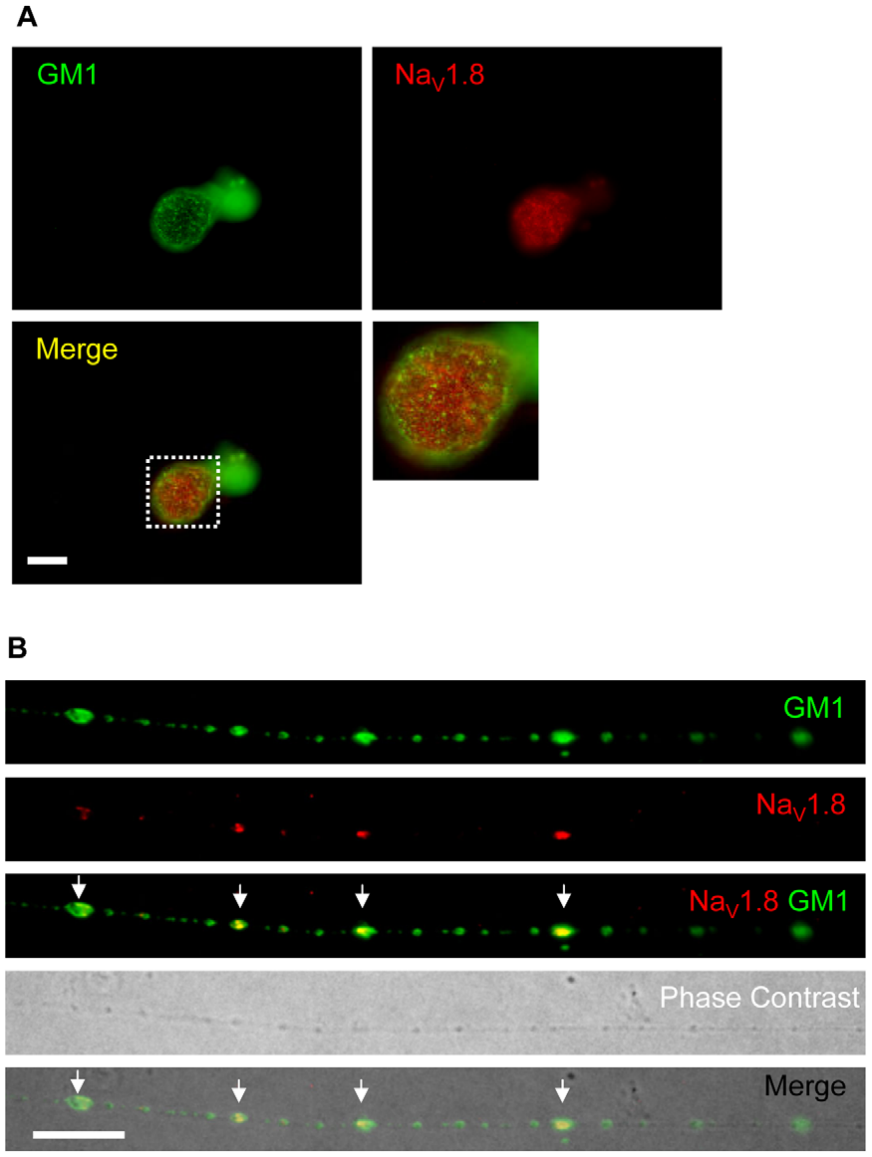
Na_V_1.8 clusters colocalise with GM1 along the axons of small DRG neurons *in vitro*. Endogenous Na_V_1.8 was immuno-localised in cultured DRG neurons after 2 DIV. GM1 molecules were detected with CTB. At the level of the cell bodies there is no clear colocalisation between endogenous Na_V_1.8 and GM1 molecules (A). In contrast, along the neurites Na_V_1.8 clusters colocalise with GM1 puncta (arrows, B). Phase contrast image in B demonstrates the integrity of the neurite. Scale bars are 20 μm.

At the sub-cellular level we found that GM1 is present as puncta on the cell surface of the cell body (Figure 3A) and along the axons of DRG neurons (Figure 3B) after two DIV. At the level of the cell bodies Na_V_1.8 and GM1, did not show a clear co-localisation; it was possible to distinguish clear puncta for GM1, but Na_V_1.8 immunoreactivity was evenly distributed and occasionally aggregated in brighter patches (Figure 3A). However, along the neurites, GM1 and Na_V_1.8 showed a convincing pattern of association. Figure 3B shows a representative intact neurite with clusters of Na_V_1.8 co-localising with GM1 puncta (arrows). It is noteworthy to highlight that the phase contrast image shows an intact morphology of the neurite and that the puncta are not associated with varicosities or bulges of the membrane. We found that the majority of Na_V_1.8 clusters co-localised with clusters of GM1 (80.5%±6.3 of Na_V_1.8 clusters positive for GM1; n = 3, total number of clusters counted  = 182) (Figure 3B, arrows).

We also used the Na_V_1.8-DsRed2 construct to further investigate the co-localisation of Na_V_1.8 with lipid rafts along the neurites. We report that the clusters of Na_V_1.8-DsRed2 also co-localised with GM1 (Figure 1C, left panel), thereby confirming the previous immunocytochemical finding with the endogenous channel, and showing that the addition of a fluorescent tag to Na_V_1.8 neither disrupts its ability to cluster nor to co-localise with GM1. Even though DsRed2 displays faster maturation compared to wild type red fluorescent protein, we confirmed that the co-localisation between GM1 and Na_V_1.8-DsRed2 is not due to the maturation of DsRed2, which may undergo a green fluorescent state (Figure S2).

Lipid rafts are heterogeneous micro-domains and two distinct types have been described: planar and caveolae-type. Flotillin-1 is enriched in planar lipid rafts [31] and in neuronal cells it is present in non caveola-type rafts [32] while Caveolin-2 is present in caveolae-type lipid rafts [33]. In order to ascertain if Na_V_1.8 shows different partitioning between planar and caveolae-type we tagged the C-terminus of Flotillin-1 and Caveolin-2 with the green photochromic fluorescent protein Dronpa [34] and delivered the plasmid DNA to DRG neurons. To analyse the degree of co-localisation between endogenous Na_V_1.8 and these lipid raft markers we detected Na_V_1.8 by immunocytochemistry. We found that endogenous Na_v_1.8 showed a higher percentage of co-localisation with Flotillin-1-Dronpa (planar rafts), compared to Caveolin-2-Dronpa (caveolae rafts) (Figure S3 A, B).

### Na_V_1.8 co-purifies with lipid rafts *in vivo* and *in vitro,* and Na_V_1.8-raft association *in vitro* is impaired by MβCD and 7KC treatments

Lipid rafts, due to their biophysical features, are resistant to non-ionic detergents at 4°C, a property that can be exploited to separate them from the soluble portion of the membrane (non-lipid raft) by ultra-centrifugation on a density gradient [16], [35], [36], [37]. Because we found that Na_V_1.8 co-localised with lipid and protein raft markers *in vitro*, we hypothesised that Na_V_1.8 may associate with lipid rafts. We therefore analysed its partition between lipid rafts and the soluble fraction from sciatic nerve *ex vivo* and cultured DRG neurons, to provide further evidence for this association.

**Figure 4:**
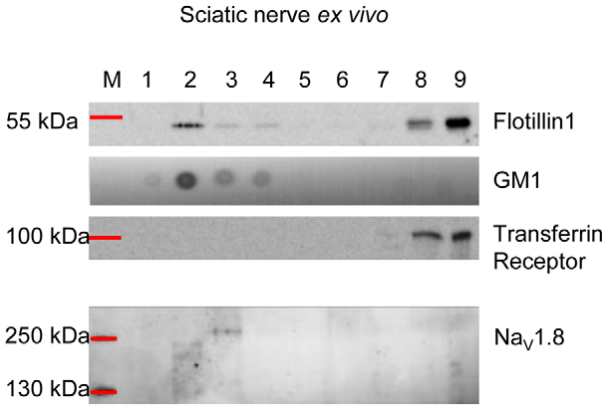
Na_V_1.8 associates with lipid rafts in the sciatic nerve. Lipid rafts were extracted from the sciatic nerve. After centrifugation on an Iodixanol density gradient, fractions were analysed by western blotting and dot blot analysis to assess lipid raft isolation and Na_V_1.8 partitioning between lipid rafts and the non-raft portions of the membrane. Flotillin1 and GM1 were used as a protein and lipid marker of lipid rafts, respectively. Transferrin receptor was used as a marker of non-raft portions. In the sciatic nerve the totality of Na_V_1.8 is associated with lipid rafts. M represents protein ladder, the recovered fractions are numbered from 1 (top fraction) to 9 (bottom fraction).

The sciatic nerve is a spinal nerve which contains the axons of sensory and motor neurons, whose cell bodies are located in the DRGs and ventral horn of the spinal cord, respectively. Since Na_V_1.8 is localised in the unmyelinated axons of the sciatic nerve [38] we tested if Na_V_1.8 exists in the lipid raft fraction of the sciatic nerve after OptiPrep gradient centrifugation. In this study, we used a protein and a lipid marker, Flotillin-1 and GM1 respectively, to define the floating, low density, raft fraction. We found that Flotillin-1 was present as two pools: one associated with the bottom fractions (lanes 8 and 9) and one associated with the top fractions (lanes 2, 3 and 4) (Figure 4). GM1 was highly enriched in the top fraction (lanes 2, 3 and 4). To define the non-lipid raft portion of the membrane we used Transferrin receptor, which is widely used as a non-raft marker [30]. Transferrin receptor was only found in the bottom fractions (lanes 8 and 9). Given the distribution of these markers, we defined fractions 2, 3 and 4 as the lipid raft fraction and fractions 8 and 9 as the soluble portion of the membrane (Figure 4). We next evaluated Na_V_1.8 partitioning in this preparation of the sciatic nerve, and remarkably Na_V_1.8 was found only in the raft fraction (lanes 2 and 3); no Na_V_1.8 was associated with the soluble fraction (Figure 4). Of note, Na_V_1.8 was present at slightly different sizes in lanes 2 and 3, which may represent different glycosylation states of the channel [39], [40], [41]. Interestingly it has been reported that different glycosylation states may act as sorting signals for raft association [42], [43], [44].

In contrast, when we performed OptiPrep gradient centrifugation with freshly extract DRG tissue, containing the nerve cell bodies, Na_V_1.8 was mostly associated with the soluble fraction and only a minor amount of Na_V_1.8 could be detected in the lipid raft fraction (data not shown). To summarise, Na_V_1.8 was mainly localised in the lipid rafts fraction of sciatic nerves, while Nav1.8 was located in the non-raft portion of the cell bodies of DRG neurons.

**Figure 5:**
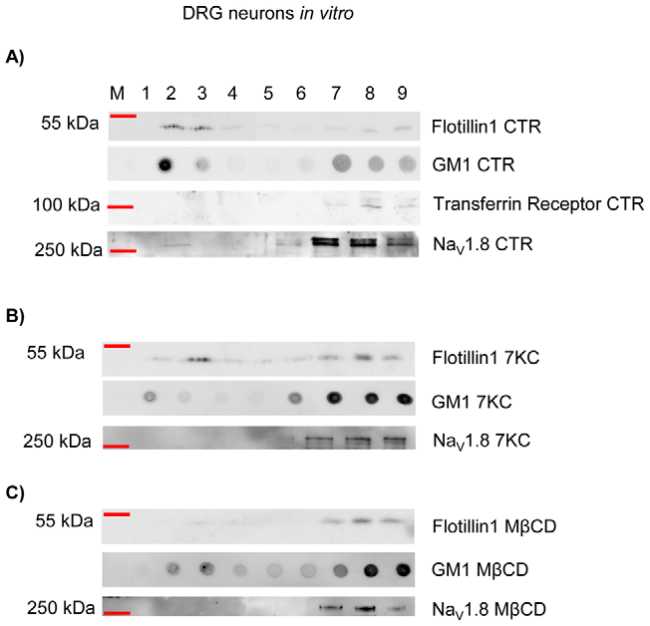
Na_V_1.8 associates with lipid rafts in DRG neurons *in vitro*. Lipid rafts were extracted from DRG neurons after 2 DIV. After centrifugation on an Iodixanol density gradient fractions were analysed by western blotting and dot blot analysis to assess lipid raft isolation and Na_V_1.8 partitioning between lipid rafts and the non-raft portions of the membrane. Flotillin1 and GM1 were used as a protein and lipid marker of lipid rafts, respectively. Transferrin receptor was used a marker of non-raft portions. Na_V_1.8 is associated with both lipid rafts and non-raft portions of the membrane (A). Incorporation of 7KC into the neuronal plasma-membranes impairs lipid raft stability. In this condition total Na_V_1.8 is associated with the non-raft portion of the membrane (B). Depletion of cholesterol from the neuronal membrane, by using MβCD, leads to lipid rafts disruption. Na_V_1.8 is only associated with the soluble, non-raft, portion of the membrane upon this treatment (C). M represents protein ladder, the recovered fractions are numbered from 1 (top fraction) to 9 (bottom fraction).

Similarly to the DRG and sciatic nerve *ex vivo* preparations, we separated lipid rafts from DRG neurons cultured for two DIV. From this source we found that the majority of Flotillin-1 was recovered from the low density fractions (Figure 5A, lanes 2 and 3). GM1 associated with top (Figure 5A, lanes 2 and 3) and bottom fractions (Figure 5A, lanes 7, 8 and 9). Transferrin receptor, as expected, did not display raft-like properties and was retained in the soluble fraction (Figure 5A, lanes 7, 8 and 9). These data clearly show that cultured DRG neurons, similar to sciatic nerve *in vivo*, contain lipid rafts which can be extracted from the soluble fraction. Therefore, from plated DRG neurons, by density gradient, lipid rafts were defined by floating fractions 2 and 3. We investigated Na_V_1.8 co-purification with rafts after two DIV and found that Na_V_1.8 was clearly associated both with the lipid raft (Figure 5A, lane 2) and the non-lipid raft (Figure 5A, lanes 7, 8 and 9) fractions.

Lipid raft integrity is dependent on the presence of cholesterol, and the depletion of cholesterol from the cell membrane by Methyl-β-cyclodextrin (MβCD) leads to their disruption. In addition, lipid rafts are liquid ordered micro-domains and the delivery of a cholesterol analogue, 7-ketocholesterol (7KC), to cell membrane has also been found to negatively affect raft stability [25], [45], [46].

For the purpose of interfering with raft integrity, we incubated DRG neurons, cultured for two DIV, with either 10 mM MβCD or 50 µM 7KC (delivered as a complex with MβCD; see the Materials and Methods) for 30 min at 37°C. MβCD and 7KC were used to deplete cholesterol from the neurons and to disrupt the lipid ordered phase of neuronal rafts, respectively. Control cells were left untreated (CTR). We assessed how raft stability and Na_V_1.8 association with rafts were affected by detergent extraction and ultracentrifugation. We found that 10 mM MβCD, and 50 µM 7KC negatively affected raft stability. In fact, upon detergent extraction, Flotillin-1 profiles between 7KC, MβCD and CTR were different. In the CTR condition (Figure 5A), the majority of Flotillin-1, as described before, was retrieved from the top fractions (lanes 2 and 3). However, in 7KC-treated neurons Flotillin-1 displayed a reduced amount on the top fractions (Figure 5B, lanes 2 and 3) and a tailing effect towards the bottom fractions. In MβCD treated samples, lipid raft disruption was also evident, with Flotillin-1 being recovered mostly from the bottom fractions (Figure 5C, lanes 7, 8 and 9) (By densitometric analysis of Flotillin-1 western blot bands we quantified the mean value of Flotillin-1 pool associated with the raft fraction in 7KC and MβCD-treated samples. Mean value expressed as % of raft associated Flotillin-1 in CTR samples ± SD; 7KC = 46.7%±24.8; MβCD = 30.9%±24.8). We also probed 7KC and MβCD treated samples for GM1. The dot blots in Figure 5B and 5C show an accumulation of GM1 in the bottom fractions and a reduction in the raft fractions, consistent with raft disruption (Figure 5B, 5C). We further investigated MβCD and 7KC effects by imaging techniques. By using the lipid phase sensitive probe di-4-ANEPPDHQ [47], [48] (Method S1) we found that both MβCD and 7KC treatments altered the lipid phase of DRG neurons, shifting it to a less ordered state, consistent with raft disruption (Figure S4). We next probed these samples for Na_V_1.8, to ascertain its partitioning upon raft depletion. Remarkably we found that, compared to CTR sample, all Na_V_1.8 was recovered from the bottom, non-raft fractions. In fact, the lipid raft-associated pool (lane 2 in CTR), was absent in both 7KC- and MβCD-treated samples (Figure 5B, C), indicating that treatment with MβCD and 7KC leads to the dissociation of Na_V_1.8 from lipid rafts (By densitometric analysis of Na_V_1.8 western blot bands we quantified the mean value of Na_V_1.8 pool associated with the raft fraction in 7KC and MβCD-treated samples. Mean value expressed as % of raft associated Na_V_1.8 in CTR samples ± SD;7KC = 5.7%±1.1; MβCD = 3.5%±0.1).

In summary, we have demonstrated that Na_V_1.8 co-purifies with lipid rafts *in vitro* and along the axons of sensory neurons *ex vivo*. We also demonstrated that after MβCD and 7KC treatments Na_V_1.8-raft association is negatively affected.

We sought to investigate if lipid raft disruption had an effect on Na_V_1.8 sub-cellular localisation. After two DIV we treated neurons with 10 mM MβCD or 50 µM 7KC for 30 min at 37°C. CTR cells were left untreated or treated with 50 µM cholesterol (CHOL). After the treatments, neurons were fixed and processed for immunofluorescence. We found that the different treatments did not induce macroscopic alteration in Na_V_1.8 distribution (Figure S5).

### Lipid raft disruption negatively affects the propagation of mechanically-induced depolarisations in DRG neurons *in vitro*

Because Na_V_1.8 mediates action potential generation and propagation in DRG neurons we developed two assays to study the effect of raft depletion and concomitant Na_V_1.8 shift to the liquid disordered phase on action potential propagation in these neurons.

**Figure 6:**
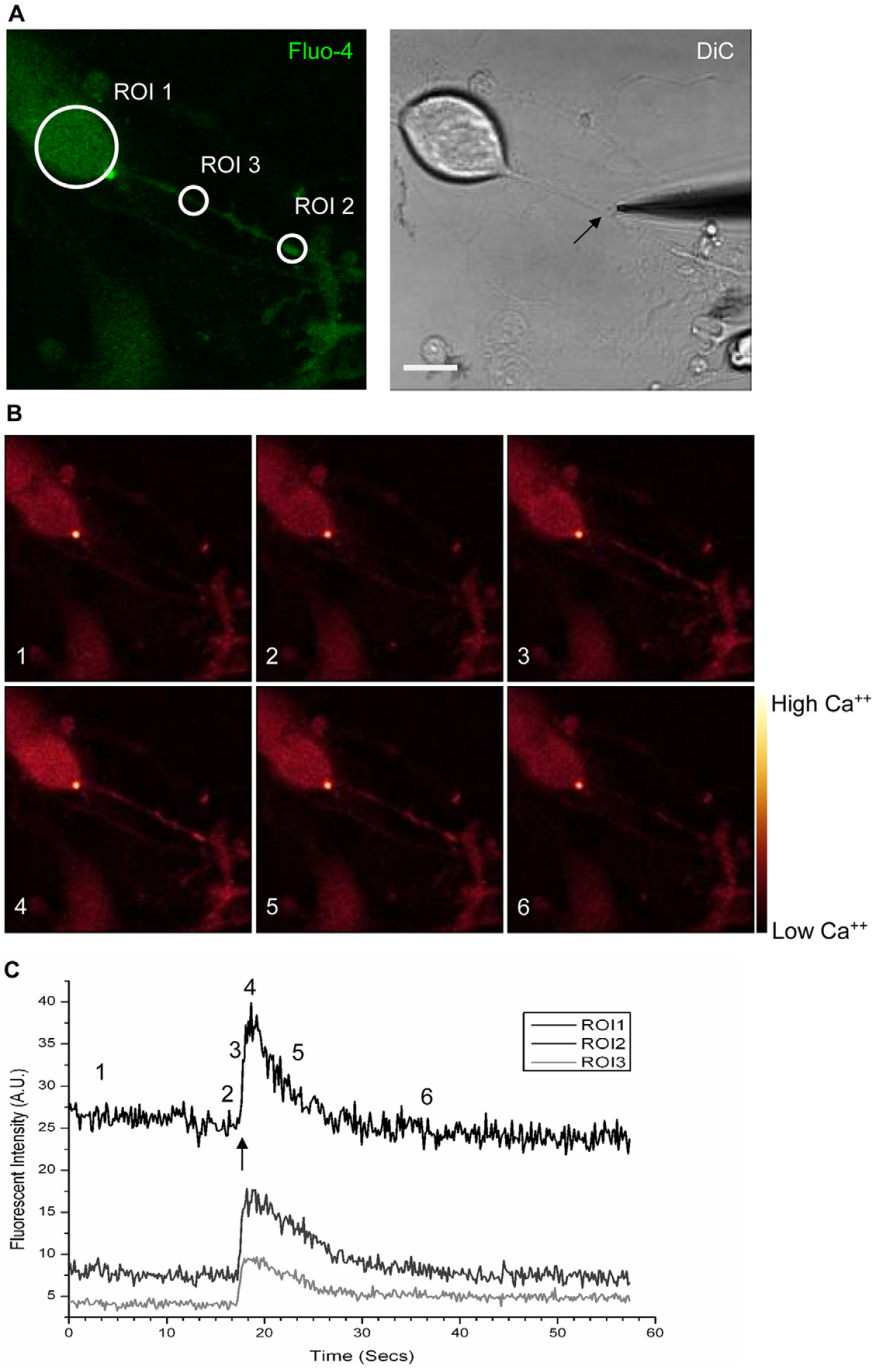
Mechanostimulation of DRG neurons *in vitro*. The figure shows a representative neuron loaded with Fluo-4 responding to a mechanical stimulus. A) Shows the Fluo-4 fluorescence and DiC image of a DRG neuron. The glass probe is visible in the DiC image, at the moment of contact with a neurite (arrow), which projects from the cell body. B) Shows the Fluo-4 fluorescence in pseudo-colour, associated with different time points during the recording. C) The graph shows the recorded fluorescence intensity of different region of interests (ROIs), visible in A. The arrow indicates the time point when the cell was stimulated; cardinal numbers refer to the time points which the images in B are associated to Scale bar is 10 µm.

We firstly developed an assay based on mechano-stimulation to study action potential propagation. Some DRG sensory neurons are mechano-sensitive *in vivo* [49], [50] and they retain the property to trigger action potentials, in response to mechanical stimuli, *in vitro* [51], [52], [53], [54], [55]. We exploited this ability to study action potential propagation in cultured DRG neurons. It has been demonstrated that real-time imaging of calcium fluxes in cell bodies of sensory neurons reliably reﬂects action potential firing patterns [56]. DRG neurons cultured for two DIV were loaded with calcium indicator Fluo-4 [57], [58]. We mechanically stimulated the neurites of the cells using a glass probe (Figure 6A, right panel), to evoke action potentials, and recorded subsequent changes in Fluo-4 fluorescent intensity in three regions of interest (ROI), ROI1 (cell soma), ROI2 (distal part of the neurite), and ROI3 (proximal part of the neurite) (Figure 6A, left panel). We found that when the glass probe contacted the neurite of a responsive neuron, an increase of Fluo-4 fluorescence was detected at the level of the neurite and this increase in fluorescence was propagated from the point of contact, in both an antidromic and orthodromic fashion, towards the end of the neurite and the cell body, respectively. When the wave of fluorescence reached the cell body, it responded with a sharp increase of fluorescence (Figure 6B). Figure 6C shows the recorded fluorescence intensity of different ROIs in a transient manner. For this series of experiments we probed axons originating from small-diameter neurons (diameter <25 µm), most likely to be nociceptive, at a mean distance of 19.8±7.6 µm from the cell soma. The increase in intracellular calcium in the cell soma, upon mechanical stimulation of the neurite, was most likely due to influx of extracellular calcium, rather than release from intracellular calcium stores, as no increase in Fluo-4 fluorescence was detected when stimulation was performed in calcium-free conditions and in the presence of 2 mM EGTA (n = 8, Table 1). To further clarify that the calcium increase in cell soma, upon axonal mechano-stimulation, was caused by membrane depolarisation propagated through VGSCs, we investigated soma responsiveness upon mechanical stimulation of the neurite in sodium-free conditions. We replaced extracellular NaCl with an equimolar amount of choline chloride to maintain correct osmolarity [59] and ionic strength of the medium. Importantly, in this condition we did not detect any somal responses upon mechanical stimulation of the axons (n = 15; Table 1). We further characterised the nature of the propagating depolarisation from the axon to the cell body, by performing the experiment in the presence of lidocaine or TTX. Given the criteria of acceptance described in the Materials and Methods, we found that, in the presence of 500 µM lidocaine, the percentage of responsive cells dropped significantly compared to control cells (% of responsive cells; CTR = 50.9%, lidocaine 500 µM = 0% *; * = p<0.05 vs. CTR. Fisher's exact test; Table 1). These data clearly indicate that VGSCs are needed for the propagation of evoked depolarisations towards the cell body and for its subsequent increase in intracellular calcium. The presence of 250 nM TTX, however, did not affect the cell responsiveness, suggesting that TTX-r VGSC channels (e.g. Na_V_1.8) are sufficient to allow the propagation of depolarisations and cell soma responsiveness (% of responsive cells; CTR = 50.9%, TTX 250 nM = 50%; Table 1).

**Table 1:** Axonal mechano-stimulation.

Treatment	n	Responsive	% Responsive neuron
CTR	55	28	50.9
Ca^++^-free	8	0	0
Na^+^-free	15	0	0
Lidocaine 500 µM	13	0	0 *
TTX 250 nM	8	4	50.0
CHOL 50 µM	24	13	54.2
7KC 50 µM	36	10	27.8 *
MβCD 10 mM	36	10	27.8 *

Effect of different compounds and lipid raft disruption on cell body responsiveness, upon axonal stimulation. Table summarises the results of axonal mechano-stimulation of DRG neurons *in vitro*. Percentages of neuronal cell bodies responsive to mechanical stimulation of the axon, in the different conditions, are listed. * = p<0.05 vs. CTR. Fisher's exact test.

We used the mechano-stimulation based assay to study action potential propagation after lipid raft disruption, a scenario in which we previously demonstrated that Na_V_1.8 shifts from the raft fraction to the soluble fraction. We treated the cells with 50 µM 7KC or 10 mM MβCD to disrupt lipid rafts. CHOL cells were treated with 50 µM cholesterol and CTR cells left untreated. In these conditions we found that cholesterol-treated cells were as responsive as control cells. Remarkably, upon lipid raft disruption and Na_V_1.8 redistribution to the soluble fraction of the cell membrane, we found a significant decrease in the number of soma able to respond to mechanical stimulation of the axon (% of responsive cells; CTR = 50.9%, n = 55; 50 µM CHOL = 54.2%, n = 24; 50 µM 7KC = 27.8%*, n = 36; 10 mM MβCD = 27.8%*, n = 36. * = p<0.05 vs. CTR using Fisher's exact test. Table 1).

**Figure 7:**
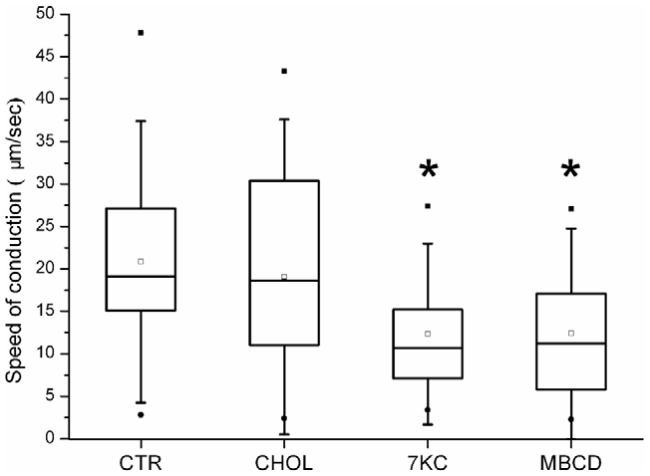
Effect of lipid raft depletion on the speed of propagation of Fluo-4 signals upon mechano-stimulation of the neurites. Box plot show that upon 7KC and MβCD treatments the speed of propagation (expressed in μm/sec) of the mechanically-evoked depolarisation is lower, compared to Control (CTR)- and Cholesterol (CHOL)-treated cells. * = p<0.05 vs. CTR. Mann-Whitney U Test.

The mechano-stimulation based assay allowed us to calculate the time between mechano-stimulation and somal response. Since the distance between the site of stimulation and the cell soma was also known, we calculated the mean speed of conduction of Fluo-4 signals, between the point of stimulation on the axon and the cell body. We found that, compared to control conditions, the mean speed of conduction was significantly lower upon lipid raft disruption. Cholesterol treatments had no effect (mean speed (in µm/sec) ± SEM: CTR = 20.8±2.1, n = 28; CHOL = 19.1±3.4, n = 13; 7KC = 12.3±2.2*, n = 10; MβCD = 12.4±2.6*, n = 10; * = p<0.05 vs. CTR., Mann-Whitney U Test; Figure 7). It should be noted that this conduction velocity is not signifying the speed of action potential propagation itself, but the speed of conduction of Fluo-4 fluorescent signals. Although this phenomenon might be dependent upon actual velocity of action potential propagation, there are also other factors involved, e.g. activation kinetics of voltage-gated calcium channels in response to membrane depolarisation, speed of Fluo-4 response to the increase of calcium concentration, and of course the property of mechano-transducers.

Since the molecular identity of mechano-transducers in DRG neurons is currently unknown [60], [61] we could not test whether they are also partitioned into lipid rafts, and thus it is unknown whether they would be affected functional by our lipid raft depletion methods. In addition, the lack of a valid positive control for mechano-stimulation does not rule out the possibility that the decrease in responsiveness of neurons, upon raft depletion, may be due to pleiotropic effects of the raft depleting agents, rather than a direct effect on the propagation of the depolarisation. Because of these reasons we have investigated mechano-stimulation of the cell bodies directly, a method of stimulation which does not involve propagation of action potentials through axons. We found that raft disruption did not alter cell responsiveness (% of responsive cells; CTR = 60.6%, n = 33; 50 µM CHOL = 61.1%, n = 18; 50 µM 7KC = 63.6%, n = 33; 10 mM MβCD = 68.4%, n = 38. p>0.5 vs. CTR. Fisher's exact test. Table 2). In our hands, direct stimulation of the cell bodies, demonstrates that both mechano-transducers and/or other calcium channels potentially involved in calcium influx, are not affected by raft disruption. This demonstrates that, upon raft depletion, the mechano-transduction mechanism and output measurement are not impaired. We also found that, in the presence of TTX, 15 minutes incubation of small-diameter DRG neurons in 22.7 mM MβCD, did not cause any obvious difference to the amplitude of sodium current densities by patch clamp recording (31.5+/−18.6 pA/pF (CTR) vs 28.5+/−15.7 pA/pF (22.7 mM MβCD)). This is clearly in agreement with the fact that Na_V_1.8 does not reside in lipid rafts in cell soma (Figure 3A), thus its function in cell soma is not affected by lipid rafts disruption.

**Table 2:** Somal mechano-stimulation.

Treatment	n	Responsive	% Responsive neuron
CTR	33	20	60.6
CHOL 50 µM	18	11	61.1
7KC 50 µM	33	21	63.6
MβCD 10mM	38	26	68.4

Effect of lipid raft disruption on cell responsiveness, upon somal stimulation Table summarises the results of somal mechano-stimulation of DRG neurons *in vitro*. Percentages of neuronal cell bodies responsive to mechanical stimulation of the soma, in the different conditions, are listed. p>0.5 vs. CTR. Fisher's exact test.

### Lipid raft disruption negatively affects the propagation of chemically induced depolarisations in DRG neurons *in vitro*

We further investigated action potential propagation along the axons of DRG neurons in control and raft depleted conditions by exploiting the properties of Campenot chambers, which allow a compartmentalisation of different parts of the neurons.

**Figure 8:**
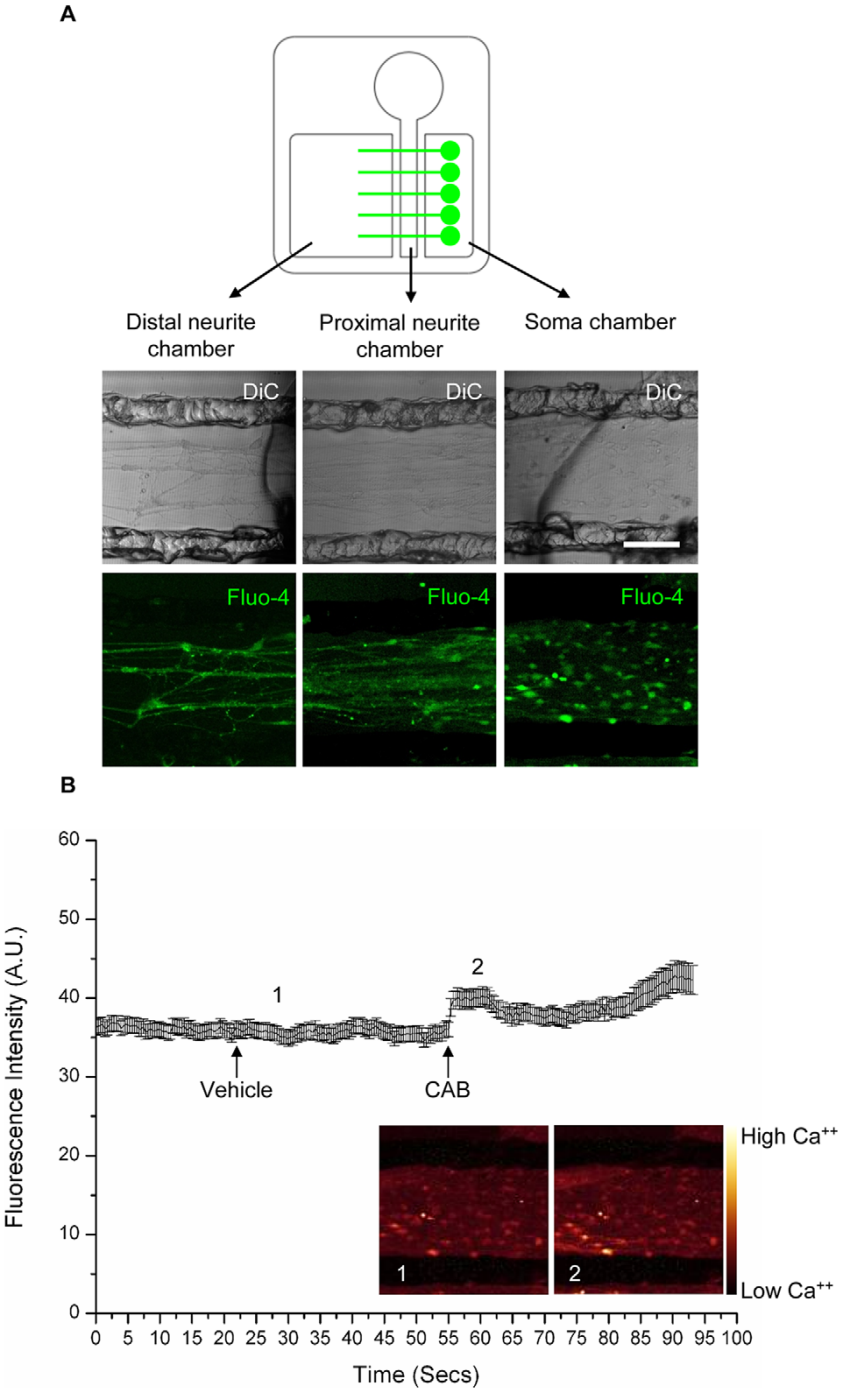
Chemical stimulation of DRG neurons: axonal stimulation and soma recording. The figure shows the effect, at the level of the cell bodies, of axonal chemical stimulation. A) Shows the schematic representation of the Campenot chamber set-up with the Fluo-4 fluorescence and DiC image of the cell bodies (Soma chamber) and neurites projecting to the “Distal neurite” chamber, through the “Proximal neurite” chamber. B) The graph shows the recorded fluorescence intensity of different cell bodies visible in A (Soma chamber). Each data point is the mean fluorescence intensity ± SEM of different cell bodies (n = 58). Images in pseudo-colour represent the “Soma chamber” Fluo-4 fluorescence. The arrows indicate the time points when Vehicle and the Capsaicin, ATP, Bradykinin (CAB) cocktail have been applied; cardinal numbers refer to the time points depicted by the pseudo-colour images. Scale bar is 100 µm.

We first developed a culture system where DRG neurons can be functionally segregated in different compartments. We used a Campenot chamber with three separate compartments, so that DRG neurons could be spatially separated into, 1) cell bodies (contained in the “soma chamber”), 2) proximal neurites (contained in the “proximal neurite chamber”), and 3) distal neurites (contained in the “distal neurite chamber”) in the culture system (Figure 8A).

To study the propagation of depolarisations, we induced action potentials at the neurite terminals in the “distal neurite chamber” by exposing the DRG nerve terminals to a cocktail of chemicals (10 μM Capsaicin, 300 μM ATP, 10 μM Bradykinin; referred to subsequently as CAB) and monitored calcium influx at the level of the cell bodies.

We found that axonal stimulation by CAB carried out in the “distal neurite chamber” was able to elicit a calcium influx that traveled through the “proximal neurite chamber” and invaded the cell bodies in the “soma chamber” (Figure 8B, time point 2). Application of vehicle did not elicit any response (Figure 8B, time point 1). We then investigated the nature of the propagating depolarisation by substituting NaCl in the “proximal neurite chamber” with equimolar choline chloride. When CAB was applied to the “distal neurite chamber”, in the absence of sodium ions in the “proximal neurite chamber”, we found that no cells responded with an increase of fluorescence, compared to control cells (Table 3). These data clearly indicate that the presence of sodium ions, in the middle part of the neurites, is required for the propagation of an action potential towards the cell bodies. We have also investigated the contribution of TTX-r currents in the propagation of action potentials. For this purpose we added 250 nM TTX to the “proximal neurite chamber” only. This concentration is known to completely block all TTX-s channels. Na_V_1.8, being TTX-r, is not blocked at this concentration [3], [5], [9], [62]. In this condition, the TTX-s channels in the “distal axonal compartment” (where stimulation is carried out) and in the “soma compartment” are not blocked. We found that the blockade of TTX-s currents, in the middle regions of axons, did not impair action potential propagation. In fact, compared to the CTR condition, the same number of cells responded to chemical stimulation (mean percentage of responsive neurons in TTX treated samples, expressed as % of CTR ± SEM = 80.3±8.9, n = 3; p = 0.15, Student's unpaired two-tailed *t*-test; Table 3). This result shows that the majority of sodium currents encoding the propagation of the action potential in the middle parts of the axons are indeed mediated by TTX-r VGSCs.

**Table 3:** Chemical stimulation.

CTR Treatment	Na^+^-free condition
100.0%	0.0%
CTR Treatment	TTX Treatment
100.0%	80.3±8.9%
CTR Treatment	CHOL Treatment
100.0%	103.0±33.6%
CTR Treatment	7KC Treatment
100.0%	36.1±13.2% *
CTR Treatment	MβCD Treatment
100.0%	36.6±10.0% *

Effect of different compounds and lipid raft disruption on cell responsiveness, upon chemical stimulation. Table shows the percentage of neurons responding to axonal chemical stimulation after the different treatments. Data are presented as means of responsive neurons expressed as % of CTR ± SEM. * = p<0.05 vs. CTR, Mann-Whitney test.

Having demonstrated that action potential propagation was mostly mediated by TTX-r currents (e.g. Na_V_1.8) in this set-up, we investigated the effect of lipid raft disruption and Na_V_1.8 shift to soluble fractions, on the propagation of action potentials. For this purpose, we disrupted lipid rafts with 50 µM 7KC or 10 mM MβCD in the “proximal neurite chamber” only and applied CAB to the axonal terminals “distal neurite chamber” and recorded fluorescence intensity at the level of the cell body “soma chamber”. Control cells were either left untreated or treated with 50 µM cholesterol (CHOL). Upon CAB application we have quantified the number of cells responsive to the chemical stimulation and found that, upon raft depletion, 7KC and MβCD treatments significantly decreased the percentage of cells responding to the chemical stimulation. On the other hand, cholesterol treatment did not cause any effect on action potential conduction (mean percentage of responsive neurons, expressed as % of CTR ± SEM; CHOL = 103.0±33.6, n = 3; 7KC = 36.1±13.2 *, n = 5; MβCD = 36.6±10.0 *, n = 4; * = p<0.01 vs CTR, Student's unpaired two-tailed *t*-test. Table 3).

## Discussion

Na_V_1.8 is a major determinant of nociceptor excitability, being responsible for the generation of action potentials in these neurons. In our hands TTX-r channels proved to be sufficient for the propagation of depolarization through the axons towards the cell bodies. The involvement of other TTX-r channels (e.g. Na_V_1.5, Na_V_1.9) can not be ruled out. Nevertheless, Na_V_1.5 is not expressed in nociceptors and Na_V_1.9 is characterised by slow activation-deactivation kinetcs [2], [6], [63], hence is not suitable for action potential conduction. Our data, combined with current knowledge of TTX-r channels, suggest that Na_V_1.8 is the most likely candidate involved in the propagation of depolarizations in nociceptors.

Previous studies have characterised its electrophysiological features and unveiled mechanisms regulating its trafficking. Nevertheless, a comprehensive description of Na_V_1.8 localisation in nociceptors is still unavailable. A more detailed knowledge of Na_V_1.8 sub-cellular localisation in nociceptors may lead to a deeper understanding of the mechanism of excitability in nociceptors.

In the present report, we have demonstrated that Na_V_1.8 is localised in clusters along the axons of unmyelinated neurons *in vitro* and *in vivo* and resides within lipid rafts. The meaning of the clustered distribution is unknown at present. The spatial localisation of ion channels is of paramount importance in shaping the electrical excitability of neuronal cells [64]. The clustered appearance of Na_V_1.8 could have a functional role in terms of action potential propagation along the axons; where this channel is expressed. One possibility is that, in unmyelinated fibres, Na_V_1.8 clusters represent sites on the membrane where action potentials can be actively generated. It is tempting to hypothesise an electrical conduction mechanism whereby, in the portion of membrane lacking Na_V_1.8, the electrical signal would spread in a manner dependent on passive cable properties, but, just before dissipating, it could be re-generated at the sites of Na_V_1.8 clustering. The close vicinity of the clusters we have found in our experiments (5–10 μm, Figure 1C) may represent a distance just short enough for the passively conducted depolarisation not to dissipate and to be regenerated and further propagated from cluster to cluster, via recruitment of Na_V_1.8. Interestingly, classic studies support a mechanism of action potential conduction in unmyelinated fibres similar to this hypothesis. Upon demyelination, sodium channels redistribute along the unmyelinated region [65], [66], [67] and electrophysiological analysis have reported that, in demyelinated axons, action potential conduction is restored before re-myelination occurs. In this condition, conduction was found to be discontinuous and proceeded via “new foci of inward membrane current”, hypothesised to be clusters of VGSCs [68]. The biological meaning of clusters could also be attributed to the fact that clusters of Na_V_1.8 increase its local concentration. It has been demonstrated that a high concentration of VGSCs is necessary for efficient action potential generation, as in the case of nodes of Ranvier, where VGSCs are highly concentrated (>1200/ µm^2^) [69], and in the axonal initial segment [70]. Another example of clusters of VGSCs at high density has been reported by Engel and Jonas. Remarkably, this study demonstrated that, in the hippocampal mossy fibre pathway, VGSCs are present at high concentration in the *en passant* boutons along the unmyelinated fibres. The authors conclude that the high density of VGSCs is needed to amplify action potentials at the pre-synaptic sites, and boost neurotransmitter release. Also, by computational modelling, it has been predicted that the clusters of VGSCs at the boutons along the axons influence the reliability and velocity of action potential propagation [71]. We have also demonstrated that mechanical and chemical stimuli are able to elicit calcium influx at the level of the cell bodies following axonal stimulation. Furthermore, cell body responsiveness depends on the presence of sodium ions in the media, it is blocked by lidocaine (a sodium channel blocker) and is unaffected by TTX (which binds to TTX sensitive channels but not to Na_V_1.8). These results points at Na_V_1.8 as the key mediator of the propagation of the mechanically- and chemically-evoked depolarisation. Further investigation with electrophysiological techniques could clarify the biological significance of Na_V_1.8 sub-cellular distribution in terms of action potential propagation in our experimental set-up and *in vivo*. In summary, since neuronal excitability is dependent on many factors, including ion channel sub-cellular localisations and local densities, Na_V_1.8 clusters may be at the base of action potential generation and conduction, and be important to shape the excitability of unmyelinated axons of nociceptors.

In the present report we also show that Na_V_1.8 resides in lipid rafts in the axons of unmyelinated fibres. We do not have information about the sorting signals that drive Na_V_1.8 to membrane rafts. Previous reports demonstrated that Na_V_1.8 must bind to chaperon protein p11 to be efficiently translocated into the neuronal membrane [24]. Also, p11 itself has been found to partition in lipid rafts [72], [73], [74]. It could be hypothesised that p11 act as a raft-sorting factor for Na_V_1.8. Other chaperon proteins such as Pdzd2 may also be responsible for Na_V_1.8 clustering in lipid rafts [75]. In addition, sodium channel sub-unit β1 has been shown to partition into lipid raft and to act as a cell adhesion molecule. Given these data it would interesting to explore the possibility that sub-unit β1 is involved in Na_V_1.8 targeting and clustering in rafts [76].

It should also be noted that a minority of Na_v_1.8 clusters co-localised with Caveolin-2, and there are two potential Caveolin binding sites within the Na_v_1.8 α-subunit.

Additional proof for Na_V_1.8 trafficking to lipid rafts came from the evidence that, in our hands, the disruption of membrane rafts correlates with Na_V_1.8 shifting to the non-raft portion of the membrane. We employed MβCD and 7KC to negatively affect raft integrity. MβCD represents the ‘gold standard’ for experimental cholesterol depletion from membrane. Indeed lipid raft integrity relies on the presence of cholesterol in the membrane; MβCD mediated cholesterol efflux determines disruption of lipid rafts [20], [77], [78], [79]. 7KC, on the other hand, modulates raft properties, and their ability to resist non-ionic detergent lysis, by decreasing the degree of order of the lipid phase [25]. 7KC differs from cholesterol only for a ketone group which protrudes perpendicularly from the sterol plane. The ketone group limits the depth of 7KC insertion into the membrane and its interaction with phospholipids acyl chains. Importantly, the alignment of the sterol ring of 7KC with trans-configured saturated acyl chains of sphingoglycolipids is impaired and this leads to decreased formation of ordered membrane domains [46]. Importantly, lipid raft depletion leads to a Na_V_1.8 shift to the soluble portion of the membrane and it correlates with impaired neuronal excitability in DRG neurons. Lipid rafts play a role in protein clustering on the membrane [28], [29]. We initially hypothesised that lipid raft integrity was necessary for the maintenance of Na_V_1.8 clusters and efficient neuronal excitability. In contrast, MβCD and 7KC did not change its distribution on the membrane, although there is a possibility that the distances between each Na_V_1.8 molecule in the clusters were widened by lipid raft disruption, but this could not be detected by the current method. Other approaches, such as electron microscopy or FRET assay, may help to reveal the changes of Na_V_1.8 location beyond the detection level of confocal microscopy. Also, it is worth underlining that we have analysed Na_V_1.8 shortly after raft depletion. It is undisputed that ion channels are tethered to the cytoskeletal protein [64], [80]. If raft had an influence on channel's clustering it could be hypothesised that the network linking Na_V_1.8 to the cytoskeleton may mask this effect upon an acute raft depletion and short term observation. An alternative hypothesis may potentially explain the failure of the propagation of the depolarisations mediated by Na_V_1.8. There is a constantly growing body of evidence which focuses on how lipid rafts directly alter the electrophysiological properties of ion channels (reviewed in [19]). Rafts are characterised by a liquid ordered phase, with different lateral pressures, viscosity and by-layer thickness, compared to non-raft regions of the membrane. These parameters can influence protein properties by modulating, for example, kinetics of transition between different conformational states (e.g. the process open-closed-inactivated in VGSCs) [77], [81], [82], [83], [84], [85]. It has been predicted, for example, that Na_V_1.6 channels conformational equilibria would differ between “bulk” non raft membranes and lipid rafts [86]. So far all electrophysiological recordings for Na_V_1.8 have been made at the cell soma level where Na_V_1.8 does not colocalise with lipid rafts. On the other hand, patch clamping of unmyelinated axonal membranes has not yet been achieved. It is, therefore, plausible that the properties (namely activation threshold) of clustered Na_V_1.8 in lipid rafts may be different from the channels reside in the cell soma membrane. Thus, it could be argued that MβCD- and 7KC-mediated raft disruption alters the biophysical property of membrane rafts, which in turn affect Na_V_1.8 electrophysiological characteristics, and ultimately cell excitability.

Lipid rafts also modulate cell signalling, by segregating or facilitating the interaction of certain molecules [36], [87], [88], [89]. A potential way rafts could influence action potential propagation is by indirectly influencing Na_V_1.8 properties. Na_V_1.8 currents are modulated by NGF, which binds to TrkA, and intracellularly by PKA and PKCε [90]. In neuronal cells, PKC and TrkA have been reported to translocate into lipid rafts upon activation and that raft integrity is required for intracellular signalling [91], [92]. In our model, a plausible hypothesis could be that raft depletion alters the signalling between TrkA, PKCε and Na_V_1.8 resulting in modified properties of Na_V_1.8. Lipid rafts have also been shown to regulate endocytosis of membrane proteins [20]. One potential explanation for failure of action potential conduction in raft-depleted samples could be that, in the absence of rafts, Na_V_1.8 is recruited into endocytotic pathways. This would lead to a reduction of TTX-r currents, which will impair impulse propagation along the axons.

We have demonstrated that TTX-s channels (e.g. Na_V_1.7 in nociceptors) do not substantially contribute to action potential propagation in our system (Table 3). Nevertheless, it may be hypothesised that Na_V_1.7 also partitions into lipid rafts and may have an indirect effect on Na_V_1.8-mediated action potential propagation. If raft depletion altered Na_V_1.7 properties, we could speculate that a defective boost of ramp currents (mediated by this channel [93]) may impair Na_V_1.8 recruitment, due to its high threshold of activation [94]. Na_V_1.9, the other TTX-r VGSC expressed in sensory neurons, is not involved in action potential generation because of its electrophysiological properties, and instead contributes to set the resting membrane potential [6]. If raft disruption altered Na_V_1.9 gating properties, a change in resting membrane potential could affect Na_V_1.8 availability to fire action potentials. These examples could be extended to other classes of proteins that contribute to membrane excitability, like Na^+^/K^+^ ATPase and leaky potassium channels. Thus, we highlight the potential importance to study different classes of proteins specifically localised in lipid rafts of nociceptors.

In conclusion, we have found that Na_V_1.8 resides in lipid rafts along the axons of DRG neurons *in vitro* and *in vivo*. Furthermore, the depletion of lipid rafts leads to Na_V_1.8 being shifted to the non-raft portion of the membrane and it correlates with impaired cell excitability. We conclude that the effect of lipid rafts on cell excitability represents a novel aspect to be considered in the efforts aiming to understand the fundamental properties of nociceptors and neuronal cells in general.

## Supporting Information

Figure S1 **Na_V_1.8 sub-cellular distribution in large-diameter neurons** ***in vitro*** **.** In large-diameter neurons, identified by morphology (A, right panel) and by the immuno-reactivity for NF200 (B, right panel), Na_V_1.8 is evenly distributed, or associated in large patches, along the neurites (A, B; arrowheads). Na_V_1.8 is also enriched in the cell somas (A, asterisk). Phase contrast images in A show the overall morphology of the neurons (neurites and cell bodies) with Na_V_1.8 immunoreactivity superimposed. Scale bars are 20 µm.
(DOCX)

Figure S2 **Na_V_1.8-DsRed2 does not display green fluorescent protein properties.** We transfected ND7-23 cells with Na_V_1.8-DsRed2 or green fluorescent protein (GFP) and imaged the cells 24 hours after transfection (Method S2). The image shows that the filter set and acquisition properties (Green channel) is suited to visualise the GFP (acting as positive control). When Na_V_1.8-DsRed2 is imaged with these settings it does not show any green fluorescence. On the contrary, Na_V_1.8-DsRed2 associated fluorescence is only visualised when the construct is imaged with settings suitable to collect red fluorescence (Red channel). ND7-23 were imaged at low (40x objective) and high (100x objective) magnification. It is worth noting that in this cell type GFP construct diffuses throughout the cell while Na_V_1.8-DsRed2 is excluded from the nucleus. Scale bars are 20 µm.
(DOCX)

Figure S3 **Na_V_1.8 preferentially colocalises with Flotillin1-Dronpa.** DRG neurons were transfected with plasmids encoding fluorescent constructs Flotillin1-Dronpa and Caveolin2-Dronpa, markers for planar and caveola type lipid rafts respectively. Endogenous Na_V_1.8 was immuno-localised after 2 DIV. A, shows a representative neuron expressing Flotillin1-Dronpa. We found that Flotillin-1-Dronpa is localised both in the soma and along the neurites of DRG neurons. This construct was evenly distributed, with brighter puncta of fluorescence along the axons (arrowheads). Interestingly, when we correlated the fluorescence of Flotillin-1-Dronpa to the localisation of endogenous Na_V_1.8, we found that Na_V_1.8 clusters showed co-localisation with the brighter spots of Flotillin-1-Dronpa along the axons (arrows). B, shows a representative neuron expressing Caveolin2-Dronpa. Caveolin-2-Dronpa showed a distinct clustered organisation along the neurites (arrowheads). In neurons with a clustered distribution of Caveolin-2-Dronpa, we found that few Na_V_1.8 clusters were associated with Caveolin-2-Dronpa puncta, with the majority being excluded from it. Scale bars are 20 μm.
(DOCX)

Figure S4 **7KC and MβCD effect on DRG neurons detected by using lipid phase sensitive probe di-4-ANEPPDHQ.** We employed imaging techniques to monitor 7KC and MβCD mediated lipid rafts disruption. We exploited the remarkable feature of fluorescent dye di-4-ANEPPDHQ to act as a sensor for the membrane lipid phase [43]. It displays a blue-shift of the emission spectrum in the liquid ordered phase (raft-like) compared to liquid disordered phase (non raft) [44]. Since MβCD and 7KC disrupt lipid rafts we hypothesised that the emission of di-4-ANEPPDHQ would be red-shifted compared to controls samples, because of a reduced liquid ordered phase (reflecting a decreased amount of liquid ordered lipid raft microdomains). We treated cells with 50 µM 7KC and 10 mM MβCD to disrupt lipid rafts; Control cells were either treated with 50 µM cholesterol (CHOL) or left untreated (CTR). To determine the effect of the compounds on the lipid phase we constructed the emission spectra of di-4-ANEPPDHQ by performing a λ scan. The graph shows the normalised emission spectra of the di-4-ANEPPDHQ bound to DRG neurons. The spectra are constructed by reading fluorescence intensity from 510 nm to 690 nm (each data point is presented as the mean fluorescence intensity ± SEM). CTR and CHOL treated samples show completely overlapping spectra, suggesting that CHOL treatmeant does not alter the phase of the membrane. On the contrary, both 7KC and MβCD determine a red-shift of the spectra, compared to CTR and CHOL treated samples (Calculated emission maxima, presented as mean emission maxima ± SEM: CTR = 590±0 nm; CHOL = 590±0 nm; 7KC = 596±1.8 nm *^,^^#^; MβCD = 596±1.6 nm *^,^^#^; * = p<0.01 vs CTR, ^#^ = p<0.01 vs CHOL. One-way ANOVA, followed by Tukey's post-hoc tests; n = 11). This result clearly indicates that both 7KC and MβCD, alter the lipid phase of the neurons, shifting it to a less ordered phase, consistent with raft disruption.
(DOCX)

Figure S5 **Lipid raft disruption does not alter Na_V_1.8-cluster distribution.** Representative images demonstrating endogenous Na_V_1.8 immuno-localised in DRG neurons after 2 DIV upon raft disruption with 7KC and MβCD. Control cells were either left untreated (CTR) or treated with cholesterol (CHOL). The images show DRG neurons with Na_V_1.8 distinct puncta along the neurites (arrows).
(DOCX)

Method S1 **Di-4-ANEPPDHQ loading and imaging in cultured DRG neurons.**
(DOCX)

Method S2 **ND7-23 culture and transfection.**
(DOCX)
