# Correction: Association between Tetrodotoxin Resistant Channels and Lipid Rafts Regulates Sensory Neuron Excitability

**DOI:** 10.1371/annotation/55db09f9-cce9-44cc-8501-5f97d1d1e6a1

**Published:** 2013-05-10

**Authors:** Alessandro Pristerà, Mark D. Baker, Kenji Okuse

In the Supporting Information section, Figure S1-S5 were omitted. The figures are available here:

Figure 1: 

Figure 2: 

Figure 3: 

Figure 4: 

Figure 5: 
