# Supplementary material for: Association between Tetrodotoxin Resistant Channels and Lipid Rafts Regulates Sensory Neuron Excitability
Source: PLoS One. 2012 Aug 1;7(8):e40079. doi: 10.1371/journal.pone.0040079 (PMC3411591; doi:10.1371/journal.pone.0040079)
Supplement: Figure S1 — NaV1.8 sub-cellular distribution in large-diameter neurons in vitro . In large-diameter neurons, identified by morphology (A, right panel) and by the immuno-reactivity for NF200 (B, right panel), NaV1.8 is evenly distributed, or associated in large patches, along the neurites (A, B; arrowheads). NaV1.8 is also enriched in the cell somas (A, asterisk). Phase contrast images in A show the overall morphology of the neurons (neurites and cell bodies) with NaV1.8 immunoreactivity superimposed. Scale bars are 20 µm. (DOCX) [file pone.0040079.s001.docx]

**Supplementary figure S1.** Na_V_1.8 sub-cellular distribution in large-diameter neurons *in vitro*.

In large-diameter neurons, identified by morphology (A, right panel) and by the immuno-reactivity for NF200 (B, right panel), Na_V_1.8 is evenly distributed, or associated in large patches, along the neurites (A, B; arrowheads). Na_V_1.8 is also enriched in the cell somas (A, asterisk). Phase contrast images in A show the overall morphology of the neurons (neurites and cell bodies) with Na_V_1.8 immunoreactivity superimposed. Scale bars are 20 μm.
