# Supplementary material for: Association between Tetrodotoxin Resistant Channels and Lipid Rafts Regulates Sensory Neuron Excitability
Source: PLoS One. 2012 Aug 1;7(8):e40079. doi: 10.1371/journal.pone.0040079 (PMC3411591; doi:10.1371/journal.pone.0040079)
Supplement: Figure S2 — NaV1.8-DsRed2 does not display green fluorescent protein properties. We transfected ND7-23 cells with NaV1.8-DsRed2 or green fluorescent protein (GFP) and imaged the cells 24 hours after transfection (Method S2). The image shows that the filter set and acquisition properties (Green channel) is suited to visualise the GFP (acting as positive control). When NaV1.8-DsRed2 is imaged with these settings it does not show any green fluorescence. On the contrary, NaV1.8-DsRed2 associated fluorescence is only visualised when the construct is imaged with settings suitable to collect red fluorescence (Red channel). ND7-23 were imaged at low (40x objective) and high (100x objective) magnification. It is worth noting that in this cell type GFP construct diffuses throughout the cell while NaV1.8-DsRed2 is excluded from the nucleus. Scale bars are 20 µm. (DOCX) [file pone.0040079.s002.docx]

**Supplementary figure S2.** Na_V_1.8-DsRed2 does not display green fluorescent protein properties. We transfected ND7-23 cells with Na_V_1.8-DsRed2 or green fluorescent protein (GFP) and imaged the cells 24 hours after transfection. The image shows that the filter set and acquisition properties (Green channel) is suited to visualise the GFP (acting as positive control). When Na_V_1.8-DsRed2 is imaged with these settings it does not show any green fluorescence. On the contrary, Na_V_1.8-DsRed2 associated fluorescence is only visualised when the construct is imaged with settings suitable to collect red fluorescence (Red channel). ND7-23 were imaged at low (40x objective) and high (100x objective) magnification. It is worth noting that in this cell type GFP construct diffuses throughout the cell while Na_V_1.8-DsRed2 is excluded from the nucleus. Scale bars are 20 μm.
