# Supplementary material for: Association between Tetrodotoxin Resistant Channels and Lipid Rafts Regulates Sensory Neuron Excitability
Source: PLoS One. 2012 Aug 1;7(8):e40079. doi: 10.1371/journal.pone.0040079 (PMC3411591; doi:10.1371/journal.pone.0040079)
Supplement: Figure S3 — NaV1.8 preferentially colocalises with Flotillin1-Dronpa. DRG neurons were transfected with plasmids encoding fluorescent constructs Flotillin1-Dronpa and Caveolin2-Dronpa, markers for planar and caveola type lipid rafts respectively. Endogenous NaV1.8 was immuno-localised after 2 DIV. A, shows a representative neuron expressing Flotillin1-Dronpa. We found that Flotillin-1-Dronpa is localised both in the soma and along the neurites of DRG neurons. This construct was evenly distributed, with brighter puncta of fluorescence along the axons (arrowheads). Interestingly, when we correlated the fluorescence of Flotillin-1-Dronpa to the localisation of endogenous NaV1.8, we found that NaV1.8 clusters showed co-localisation with the brighter spots of Flotillin-1-Dronpa along the axons (arrows). B, shows a representative neuron expressing Caveolin2-Dronpa. Caveolin-2-Dronpa showed a distinct clustered organisation along the neurites (arrowheads). In neurons with a clustered distribution of Caveolin-2-Dronpa, we found that few NaV1.8 clusters were associated with Caveolin-2-Dronpa puncta, with the majority being excluded from it. Scale bars are 20 μm. (DOCX) [file pone.0040079.s003.docx]

**Supplementary figure S3.** Na_V_1.8 preferentially colocalises with Flotillin1-Dronpa.

DRG neurons were transfected with plasmids encoding fluorescent constructs Flotillin1-Dronpa and Caveolin2-Dronpa, markers for planar and caveola type lipid rafts respectively. Endogenous Na_V_1.8 was immuno-localised after 2 DIV. A, shows a representative neuron expressing Flotillin1-Dronpa. We found that Flotillin-1-Dronpa is localised both in the soma and along the neurites of DRG neurons. This construct was evenly distributed, with brighter puncta of fluorescence along the axons (arrowheads). Interestingly, when we correlated the fluorescence of Flotillin-1-Dronpa to the localisation of endogenous Na_V_1.8, we found that Na_V_1.8 clusters showed co-localisation with the brighter spots of Flotillin-1-Dronpa along the axons (arrows). B, shows a representative neuron expressing Caveolin2-Dronpa. Caveolin-2-Dronpa showed a distinct clustered organisation along the neurites (arrowheads). In neurons with a clustered distribution of Caveolin-2-Dronpa, we found that few Na_V_1.8 clusters were associated with Caveolin-2-Dronpa puncta, with the majority being excluded from it. Scale bars are 20 μm.
