# Supplementary material for: Association between Tetrodotoxin Resistant Channels and Lipid Rafts Regulates Sensory Neuron Excitability
Source: PLoS One. 2012 Aug 1;7(8):e40079. doi: 10.1371/journal.pone.0040079 (PMC3411591; doi:10.1371/journal.pone.0040079)
Supplement: Figure S4 — 7KC and MβCD effect on DRG neurons detected by using lipid phase sensitive probe di-4-ANEPPDHQ. We employed imaging techniques to monitor 7KC and MβCD mediated lipid rafts disruption. We exploited the remarkable feature of fluorescent dye di-4-ANEPPDHQ to act as a sensor for the membrane lipid phase [43]. It displays a blue-shift of the emission spectrum in the liquid ordered phase (raft-like) compared to liquid disordered phase (non raft) [44]. Since MβCD and 7KC disrupt lipid rafts we hypothesised that the emission of di-4-ANEPPDHQ would be red-shifted compared to controls samples, because of a reduced liquid ordered phase (reflecting a decreased amount of liquid ordered lipid raft microdomains). We treated cells with 50 µM 7KC and 10 mM MβCD to disrupt lipid rafts; Control cells were either treated with 50 µM cholesterol (CHOL) or left untreated (CTR). To determine the effect of the compounds on the lipid phase we constructed the emission spectra of di-4-ANEPPDHQ by performing a λ scan. The graph shows the normalised emission spectra of the di-4-ANEPPDHQ bound to DRG neurons. The spectra are constructed by reading fluorescence intensity from 510 nm to 690 nm (each data point is presented as the mean fluorescence intensity ± SEM). CTR and CHOL treated samples show completely overlapping spectra, suggesting that CHOL treatmeant does not alter the phase of the membrane. On the contrary, both 7KC and MβCD determine a red-shift of the spectra, compared to CTR and CHOL treated samples (Calculated emission maxima, presented as mean emission maxima ± SEM: CTR = 590±0 nm; CHOL = 590±0 nm; 7KC = 596±1.8 nm *, #; MβCD = 596±1.6 nm *, #; * = p<0.01 vs CTR, # = p<0.01 vs CHOL. One-way ANOVA, followed by Tukey's post-hoc tests; n = 11). This result clearly indicates that both 7KC and MβCD, alter the lipid phase of the neurons, shifting it to a less ordered phase, consistent with raft disruption. (DOCX) [file pone.0040079.s004.docx]

**Supplementary figure S4.** 7KC and MβCD effect on DRG neurons detected by using lipid phase sensitive probe di-4-ANEPPDHQ. We employed imaging techniques to monitor 7KC and MβCD mediated lipid rafts disruption. We exploited the remarkable feature of fluorescent dye di-4-ANEPPDHQ to act as a sensor for the membrane lipid phase [43]. It displays a blue-shift of the emission spectrum in the liquid ordered phase (raft-like) compared to liquid disordered phase (non raft) [44]. Since MβCD and 7KC disrupt lipid rafts we hypothesised that the emission of di-4-ANEPPDHQ would be red-shifted compared to controls samples, because of a reduced liquid ordered phase (reflecting a decreased amount of liquid ordered lipid raft microdomains). We treated cells with 50 μM 7KC and 10mM MβCD to disrupt lipid rafts; Control cells were either treated with 50 μM cholesterol (CHOL) or left untreated (CTR). To determine the effect of the compounds on the lipid phase we constructed the emission spectra of di-4-ANEPPDHQ by performing a λ scan.

The graph shows the normalised emission spectra of the di-4-ANEPPDHQ bound to DRG neurons. The spectra are constructed by reading fluorescence intensity from 510 nm to 690 nm (each data point is presented as the mean fluorescence intensity ± SEM). CTR and CHOL treated samples show completely overlapping spectra, suggesting that CHOL treatmeant does not alter the phase of the membrane. On the contrary, both 7KC and MβCD determine a red-shift of the spectra, compared to CTR and CHOL treated samples (Calculated emission maxima, presented as mean emission maxima ± SEM: CTR= 590 ± 0 nm; CHOL= 590 ± 0 nm; 7KC= 596 ± 1.8 nm *^,^ ^#^; MβCD= 596 ± 1.6 nm *^,^ ^#^; *=p < 0.01 vs CTR, ^#^=p < 0.01 vs CHOL. One-way ANOVA, followed by Tukey’s post-hoc tests; n=11). This result clearly indicates that both 7KC and MβCD, alter the lipid phase of the neurons, shifting it to a less ordered phase, consistent with raft disruption.
