# Supplementary material for: Association between Tetrodotoxin Resistant Channels and Lipid Rafts Regulates Sensory Neuron Excitability
Source: PLoS One. 2012 Aug 1;7(8):e40079. doi: 10.1371/journal.pone.0040079 (PMC3411591; doi:10.1371/journal.pone.0040079)
Supplement: Figure S5 — Lipid raft disruption does not alter NaV1.8-cluster distribution. Representative images demonstrating endogenous NaV1.8 immuno-localised in DRG neurons after 2 DIV upon raft disruption with 7KC and MβCD. Control cells were either left untreated (CTR) or treated with cholesterol (CHOL). The images show DRG neurons with NaV1.8 distinct puncta along the neurites (arrows). (DOCX) [file pone.0040079.s005.docx]

**Supplementary figure S5.** Lipid raft disruption does not alter Na_V_1.8-cluster distribution.

Representative images demonstrating endogenous Na_V_1.8 immuno-localised in DRG neurons after 2 DIV upon raft disruption with 7KC and MβCD. Control cells were either left untreated (CTR) or treated with cholesterol (CHOL). The images show DRG neurons with Na_V_1.8 distinct puncta along the neurites (arrows).
