# Supplementary material for: Association between Tetrodotoxin Resistant Channels and Lipid Rafts Regulates Sensory Neuron Excitability
Source: PLoS One. 2012 Aug 1;7(8):e40079. doi: 10.1371/journal.pone.0040079 (PMC3411591; doi:10.1371/journal.pone.0040079)
Supplement: Method S1 — Di-4-ANEPPDHQ loading and imaging in cultured DRG neurons. (DOCX) [file pone.0040079.s006.docx]

**Di-4-ANEPPDHQ loading and imaging in cultured DRG neurons.**

Di-4-ANEPPDHQ (Invitrogen) was dissolved in 96% EtOH to a final concentration of 0.2 mM and stored at 4°C in the dark. DRG neurons were left untreated or treated with 7KC, cholesterol and MβCD as previously described. After the different treatments cells were washed and incubated with 4 μM di-4-ANEPPDHQ for 10 min at 37°C in the dark. Cells were imaged under a Leica SP5 inverted confocal microscope with a 20x air objective. The dye was excited with a 476 nm Argon laser line. Emission spectra were constructed by performing a λ scan. The fluorescence intensity was recorded across the visible spectrum in the range 510-690 nm with 19 readings. Fluorescence intensities were automatically plotted against the respective range of the spectrum they were recorded from to build the emission spectra at the end of the scan. Emission maxima was defined as the wavelength associated with the highest fluorescence intensity measured in the 19 readings.
