# Supplementary material for: Association between Tetrodotoxin Resistant Channels and Lipid Rafts Regulates Sensory Neuron Excitability
Source: PLoS One. 2012 Aug 1;7(8):e40079. doi: 10.1371/journal.pone.0040079 (PMC3411591; doi:10.1371/journal.pone.0040079)
Supplement: Method S2 — ND7-23 culture and transfection. (DOCX) [file pone.0040079.s007.docx]

**ND7-23 culture and transfection.**

ND7/23 clone is a DRG-like cell line. We cultured the cell line in DMEM with 10% FBS in 10 cm diameter dishes. The cell line was trypsinised and sub-cultured at a 90% confluence. When transfection was needed, 15,000 cells were seeded onto poly-L-Lys covered glass coverslips the day before transfection. On the day of transfection plasmid DNA (1 µg) was transfected using Lipofectamine2000 (Invitrogen) following manufacturer’s instruction.
