# Supplementary figures and images for: Correction: Association between Tetrodotoxin Resistant Channels and Lipid Rafts Regulates Sensory Neuron Excitability
Source: PLoS One. 2013 May 10;8(5):10.1371/annotation/55db09f9-cce9-44cc-8501-5f97d1d1e6a1. doi: 10.1371/annotation/55db09f9-cce9-44cc-8501-5f97d1d1e6a1 (PMC3651323; doi:10.1371/annotation/55db09f9-cce9-44cc-8501-5f97d1d1e6a1)

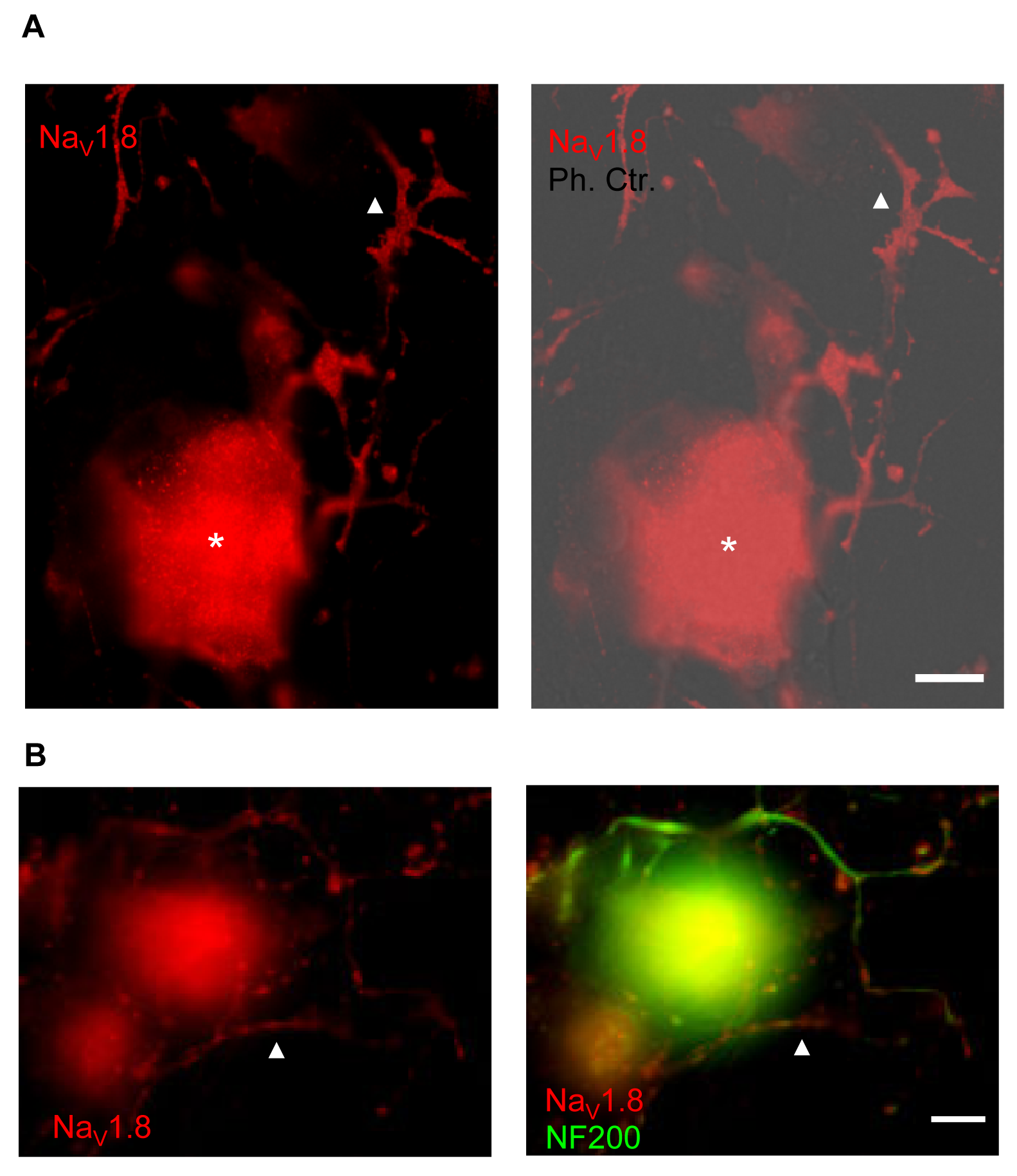

Supplement: Supplementary file 1 [file pone.55db09f9-cce9-44cc-8501-5f97d1d1e6a1.s001.tif]

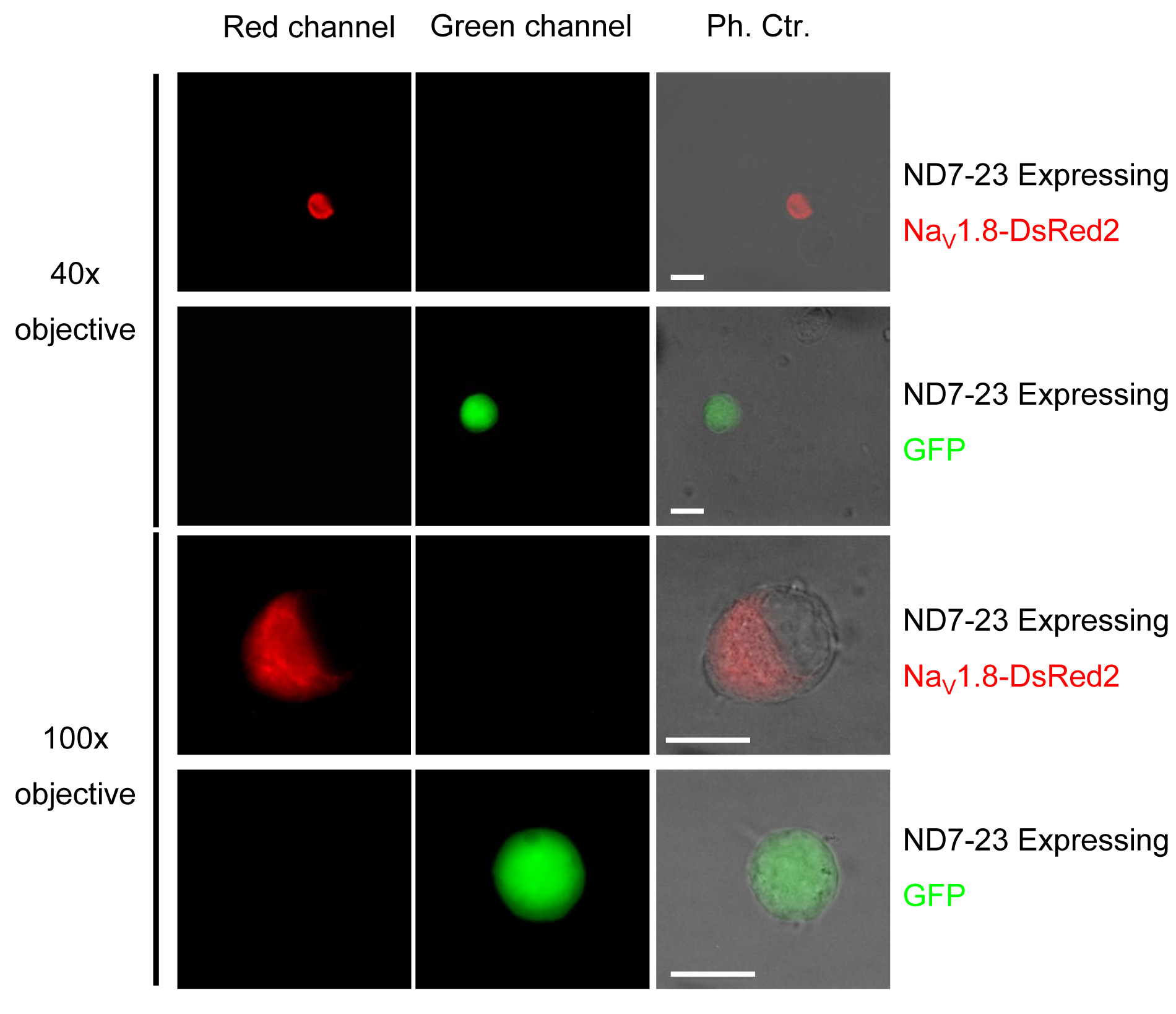

Supplement: Supplementary file 2 [file pone.55db09f9-cce9-44cc-8501-5f97d1d1e6a1.s002.tif]

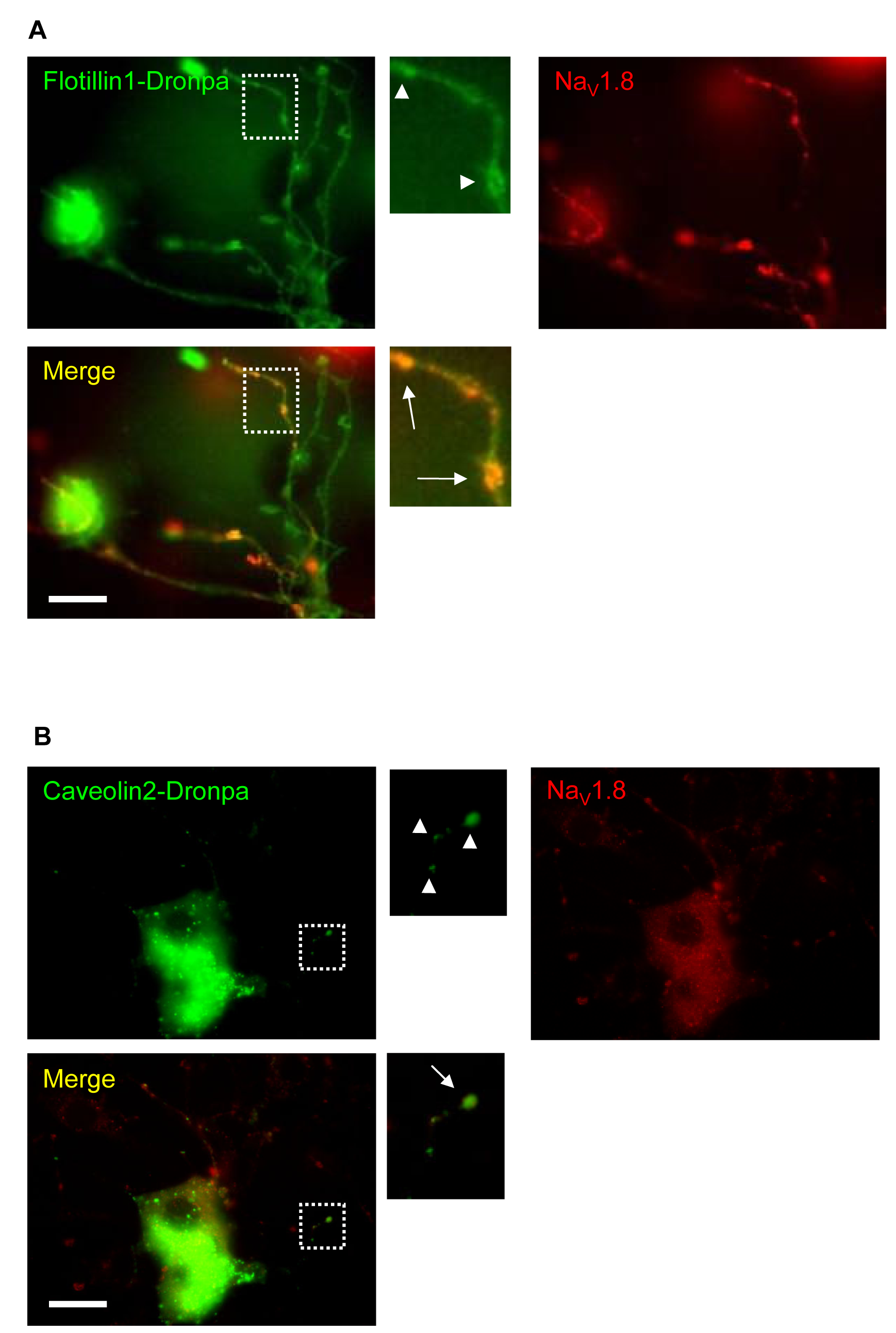

Supplement: Supplementary file 3 [file pone.55db09f9-cce9-44cc-8501-5f97d1d1e6a1.s003.tif]

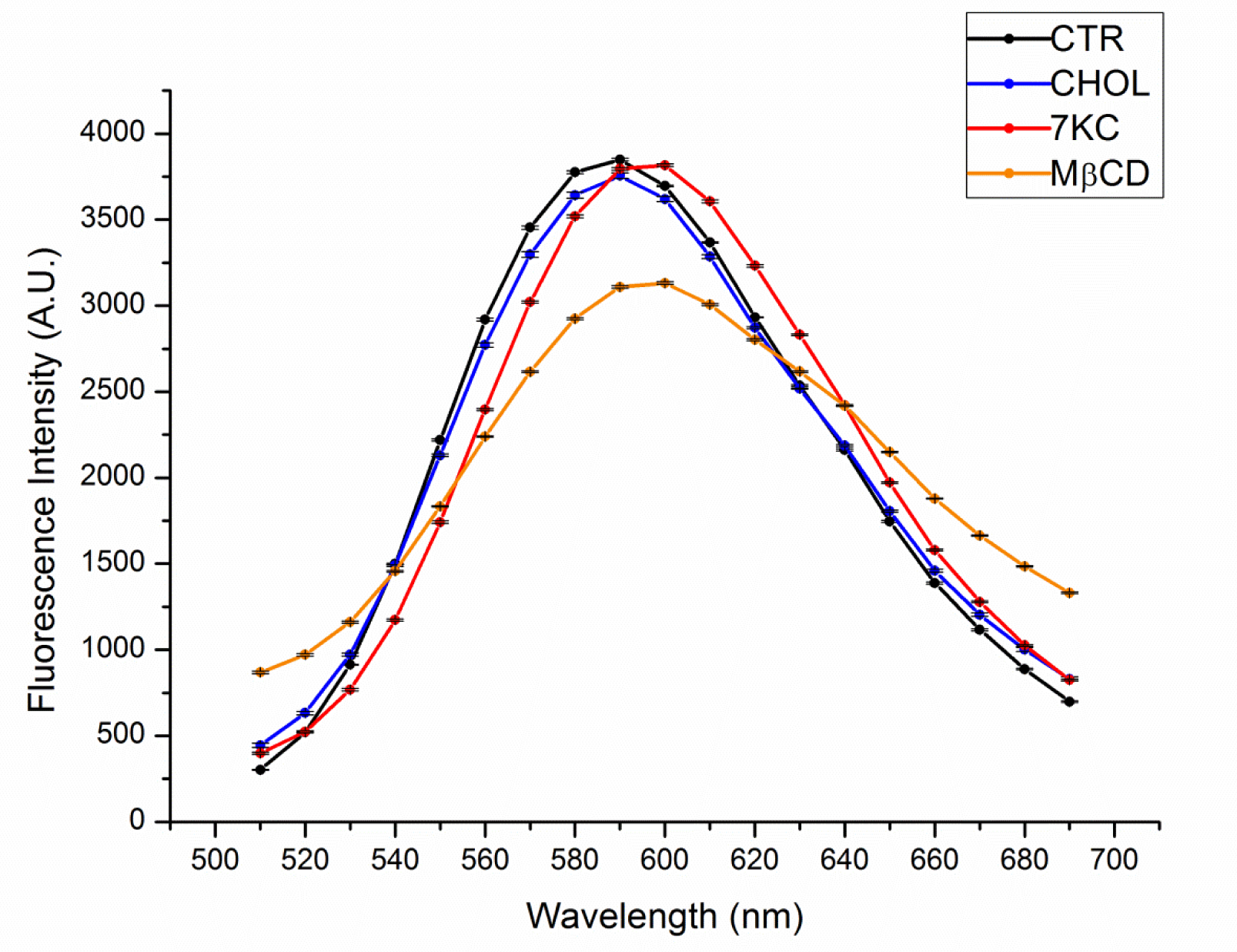

Supplement: Supplementary file 4 [file pone.55db09f9-cce9-44cc-8501-5f97d1d1e6a1.s004.tif]

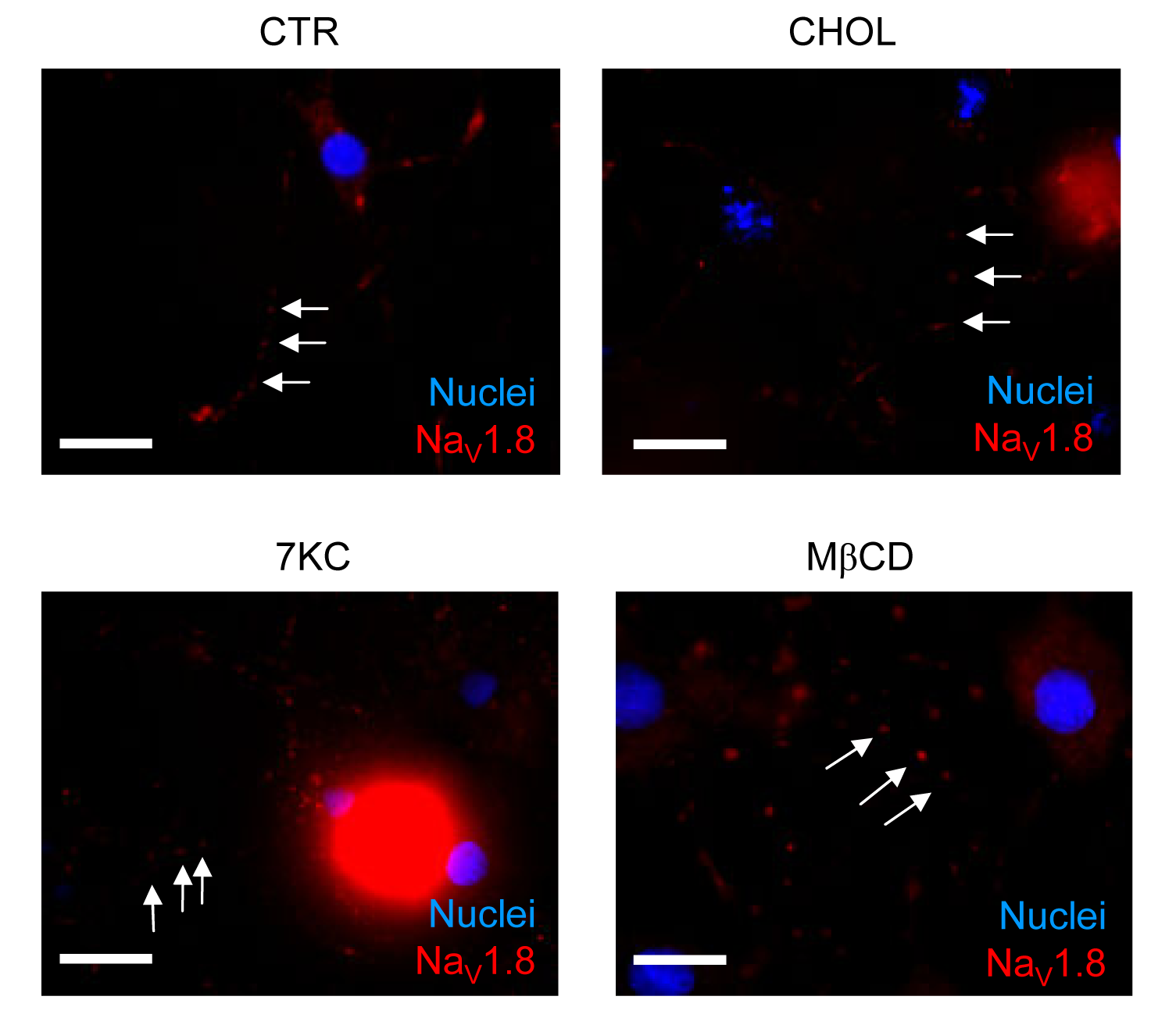

Supplement: Supplementary file 5 [file pone.55db09f9-cce9-44cc-8501-5f97d1d1e6a1.s005.tif]
